# Implantable ocular therapeutic systems: an insight into their clinical potential in the long-term treatment of ocular diseases

**DOI:** 10.1088/1758-5090/ae49fe

**Published:** 2026-03-16

**Authors:** Hyeonji Kim, Nae-Won Kang, Wen Hong, Sajjad Abdollahramezani, Gia-Han Ngo, Daanyal Raja, Olivia Peters, Vinit B Mahajan, David Myung, Charles DeBoer

**Affiliations:** 1 Department of Ophthalmology, School of Medicine, Byers Eye Institute at Stanford University, Palo Alto, CA 94304, United States of America; 2 College of Pharmacy, The Catholic University of Korea, Bucheon-si, Gyeonggi-do 14662, Republic of Korea; 3 Molecular Surgery Laboratory, Stanford University, Palo Alto, CA 94304, United States of America; 4 Department of Chemical Engineering, Stanford University, Palo Alto, CA 94305, United States of America; 5 VA Palo Alto Health Care System, Palo Alto, CA 94304, United States of America

**Keywords:** ophthalmic device, drug delivery, eye implant, clinical trial, ocular therapeutic system, translational medicine

## Abstract

Despite the rapid pace of biomedical engineering research, translating developed products into clinical practice remains challenging due to regulations, manufacturing, and long-term *in vivo* safety. The eye offers advantageous features to lower translational hurdles, making it an ideal clinical target and an approachable testbed for biofabricated implants. However, eyes also have anatomical and physiological barriers that hinder conventional ophthalmic delivery routes, leading to poor drug bioavailability. Advances in biofabrication and biomaterials used in ophthalmic therapeutic implants have the potential to address the current challenges. This review will explore biomaterials, biofabrication methods, and possible ocular implantation sites from the perspective of developing effective therapeutic implants. It also examines clinically available products and current clinical trials, along with recent advancements and next-generation technologies in ophthalmic therapeutic delivery implants. This review aims to provide insights that facilitate the translation of emerging ocular therapeutics into clinically available treatments.

## 1. Introduction

The field of biomedical engineering, particularly in biofabrication and biomaterials, has developed and introduced numerous effective therapeutic implants such as Apligraf^®^ (Organogenesis Inc.), a bioengineered skin substitute, and XIENCE^®^ (Abbott), a drug-eluting coronary stent [1, 2]. Although the research has progressed rapidly, only a few products have been clinically approved and widely adopted in the real world. Practical hurdles, such as regulatory approval, scalable manufacturing, and long-term *in vivo* safety, make translating these technologies difficult [3]. To overcome these obstacles, one must identify areas where translation is most practical.

The eye is a strong candidate organ due to its physical accessibility for direct implantation, real-time monitoring, and localized interventions [4] (figure 1). In cases where complications arise, implants can be directly surgically removed. Ocular immune privilege also reduces the risk of adverse reactions to implants [5]. These features make the eye not only an ideal clinical target but also an excellent testbed for biofabricated implants and engineered therapeutic delivery systems.

With the rising prevalence of ocular diseases such as glaucoma, age-related macular degeneration (AMD), and diabetic retinopathy, there remains an unmet global demand for effective ocular therapies [5]. This is further underscored by the shortcomings of current clinical treatment options. Conventional delivery routes—topical drops, injections, or oral/systemic administration—are hampered by the eye’s protective anatomical and physiological barriers [6]. Features like tear turnover, corneal epithelium, blood–retinal barriers, tear drainage, and blinking, lower drug bioavailability. Less than 5% of administered drugs reach therapeutic levels in ocular tissues [7]. Consequently, frequent dosing or highly concentrated drug formulations are required to treat these diseases, both of which increase the risk of side effects, ocular toxicity, and place more strain on the patient [7]. Ocular drug delivery implants can achieve therapeutic drug levels in specific ocular tissues that are otherwise inaccessible through conventional methods, bypassing anatomical and physiological barriers.

Drug delivery implants enable sustained release of drugs at a target site, minimizing systemic complications [8]. Ocular implants also offer several pharmacokinetic advantages, such as higher half-lives and lower clearance rates, improving the sustained treatment of chronic ocular diseases that require long-term management [9]. To achieve these benefits, precise release control, mechanical stability, and tissue integration are necessary, all of which rely on advanced biofabrication techniques.

Recent biofabrication advances have shown the potential for various implant designs with tailored release kinetics and integration with host tissues [4, 5, 10–12]. These strategies facilitate sustained, localized drug delivery, improved biocompatibility, and reproducible manufacturing for clinical use.

This review presents a device-centered perspective of implantable systems for ocular therapy. It outlines representative device fabrication approaches, where they reside, how they function over time, as well as their clinical use and current directions in research (figure 2). Rather than cataloging specific drugs, we focus on general design principles and translational considerations drawn from advances in biofabrication, materials, and biomedical engineering. The sections that follow provide a high-level overview of the field, including promising trends and current challenges that can inform future clinical impact within ocular therapeutics. This review especially addresses failures and bottlenecks across implant types while also providing practical insights with the aim of guiding biomedical engineers toward promising and clinically relevant directions for future work across all disciplines.

## 2. Synthetic materials for ocular therapeutic implants

Polymeric biomaterials have been at the heart of vision-restoring technology since the first intraocular lens (IOL) was introduced in 1949 [13, 14]. Today, they form the basis of ophthalmic devices and implants for injection-free drug delivery. The success of these materials hinges on their biocompatibility, drug-release mechanism, mechanical strength, and optical clarity [15–17]. Broadly, these biomaterials can be categorized into two different classes based on their degradation: non-biodegradable and biodegradable. The selection of each of these material platforms involves a trade-off between surgical burden, treatment duration, and precision of drug delivery. Here, we review some key synthetic polymers (figure 3) by discussing their properties, fabrication methods, and the advantages and challenges each one presents for ophthalmic use.

### 2.1. Non-biodegradable materials

Non-biodegradable materials are designed for long-term residence with minimal performance degradation and sustained drug delivery over extended durations, often spanning months to years [17, 18]. Ophthalmic devices incorporating non-biodegradable materials can be removed at any time if issues (e.g. inflammation) arise due to drug incompatibility or implantation error. Additionally, there is the potential for drug refilling, as the frame or reservoir can be maintained *in vivo*, providing an opportunity to adjust treatment plans more effectively. This allows for timely interventions as needed. However, their non-biodegradable nature requires surgery for both implantation and removal, subjecting patients to multiple procedures with associated risks. Some key non-biodegradable materials include polymethyl methacrylate (PMMA), polypropylene (PP), silicone, as well as polyvinyl alcohol (PVA) and poly (2-hydroxyethyl methacrylate) (PHEMA). PVA and PHEMA are commonly used as hydrogels which are water-swollen networks of cross-linked polymer chains. Below, we discuss each of these materials and how they are processed for ocular devices.

#### 2.1.1. PMMA

As an acrylic thermoplastic with a high glass transition temperature (*T*_g_) around 105 °C–113 °C, PMMA was used in the first intraocular lens [13, 14]. It exhibits a high elastic modulus on the order of a few GPa (e.g. Young’s modulus ~2 GPa), and minimal water uptake (0.4%–0.8%). PMMA is optically transparent across the visible spectrum with a refractive index of ~1.49 [13]. It is a biocompatible material with minimal chronic inflammation. PMMA devices are typically fabricated by polymerization in molds. Due to the high-temperature nature of this fabrication process, incorporating heat-degradable drugs during manufacturing is generally impossible. Thus, PMMA can be employed as an inert structural material allowing for adding drugs via surface coatings. PMMA is also somewhat hydrophobic (contact angle ~65–71°) which can cause adherence to ocular tissues if mishandled. These limitations make PMMA a less-commonly used material platform as a drug-carrier matrix, but it remains important for coatings and structural roles in ocular devices [15].

#### 2.1.2. PP

PP is a biocompatible, inert, and high-strength semicrystalline polyolefin mainly used for structural applications such as sutures (e.g. PP sutures Prolene^®^ [19]) and device support. It has a relatively high Young’s modulus (typically ~1–2 GPa), tensile strength in the tens of MPa range, and absorbs virtually no water. PP-based ocular components are fabricated by melt extrusion (e.g. extruding and drawing PP into thin fibers for sutures) or molding (e.g. IOL haptics). As processing temperatures are in the range of 160 °C, direct embedding of therapeutic drugs during fabrication is impractical [19]. Its hydrophobic nature makes PP a rarely used matrix for drug delivery, although it can be used as a physical support for drug-loaded hydrogels. PP is opaque or translucent due to its crystallinity, so it cannot be used in optical elements with transparency requirements. Overall, PP can play major supportive roles in ophthalmology (suture threads, haptic loops, capsular tension rings, etc) [20].

#### 2.1.3. Silicone elastomer

Silicone in ophthalmology typically refers to medical-grade polydimethylsiloxane (PDMS) elastomers or other silicone polymers. The silicon-oxygen backbone provides high flexibility with soft and elastomeric properties, including an extremely low *T*_g_ (≈−120 °C) [21, 22]. With Young’s modulus on the order of hundreds of kPa to a few MPa, and tensile strength around a couple of MPa (2–7 MPa, depending on the crosslink density), silicone devices can be folded or stretched without cracking. Silicone can be made highly transparent [23]. The refractive index varies between 1.4–1.55 [24], making it suitable for ophthalmic devices and lenses. Silicone is hydrophobic (contact angle ~97–120°), absorbs negligible water (~0.3%–0.4%), and is chemically inert and heat-resistant [21, 22], making it stable in the eye. Silicone elastomers can be supplied as two-part liquid polymers that crosslink even at room temperature. Casting or compression molding can be used followed by heat-curing (often at ~100 °C or lower, depending on the formulation) to fabricate ocular devices. Such moderate curing temperatures permit the integration of therapeutic agents. A silicone matrix can entrap lipophilic drugs before curing, enabling sustained drug release in the eye. One challenge, however, is controlling the release rate as highly soluble drugs may have a burst release profile. To overcome this, a surface coating or rate-limiting membrane can be added to flatten the release kinetics. One such example, the FDA-approved Retisert^®^ implant for uveitis, contains a fluocinolone acetonide tablet encapsulated by a rate-limiting silicone/PVA membrane [15], promoting drug diffusion over ~2.5 years. Silicone rubber segments have also been used in glaucoma (e.g. drainage devices) thanks to their flexibility and bio-inert properties, as well as in scleral buckles. 3D printing of silicones has emerged as a fabrication process for customized ocular devices. Overall, silicone elastomers remain a competitive material platform for ophthalmic devices that require softness, elasticity, and clarity, ranging from foldable lenses to long-term drainage implants.

#### 2.1.4. PVA

PVA is a hydrophilic polymer that can form stable hydrogels via crosslinking. One common method of creating a PVA hydrogel involves freeze–thaw cycling that induces physical crosslinks in the polymer, resulting in a flexible, elastic hydrogel with high water content (>70%–80% [25]). It is also possible to chemically crosslink PVA hydrogels using glutaraldehyde, but physical crosslinking is considered to be safer since many chemical crosslinkers can have biocompatibility issues. Since PVA hydrogels are usually processed in an aqueous solution at low temperatures, drugs can either be mixed into the solution before gelation or later absorbed into the porous hydrogel network. PVA-based device fabrication often involves casting an aqueous PVA solution in a mold, followed by crosslinking and rinsing. PVA hydrogels are highly transparent with a refractive index of ~1.33–1.35, like that of water. They can be mechanically resilient (depending on crosslink density and water content) with tensile strengths on the order of 1–2 MPa and elongations exceeding 100% [26, 27]. Generally, more freeze–thaw cycles (or chemical crosslinks) yield a stronger but less elastic hydrogel. PVA hydrogels can have an elastic modulus in the range of 0.1–0.5 MPa and reach 1–2 MPa after optimization [26, 27]. One of the first FDA-approved intraocular drug implants, Vitrasert^®^, leveraged PVA as a permeable matrix controlling the diffusion of ganciclovir, an antiviral drug released over ~58 months [26, 28]. Soft hydrogel contact lenses based on PVA (e.g. nelfilcon A) have also been used to deliver drugs by soaking the lenses and later releasing the drug into the tear film. Due to their high-water content, drug diffusion is relatively fast. PVA hydrogels are prone to an initial drug release burst, although it was shown that modifying PVA’s hydrogels network or adding hydrophobic domains can mitigate this issue [29, 30]. PVA hydrogels also exhibit biocompatibility and conformability with minimal irritation. Despite a series of advantages, crosslinked PVA is nondegradable and must be removed if long-term implantation is not desired. It is also limited by its weak mechanical strength.

#### 2.1.5. Poly(2-hydroxyethyl methacrylate) (PHEMA)

PHEMA is an established biocompatible hydrogel polymer first used in early soft contact lenses. A typical low-water PHEMA lens is approximately 38% water, has a *T*_g_ of approximately 12 °C–15 °C (soft at room and body temperature) [31, 32], and an elastic modulus around 0.4–0.8 MPa. 2-hydroxyethyl methacrylate (HEMA) hydrogels feature a higher water content (50%–70% water) and are even softer (modulus ~0.1–0.3 MPa) but less durable. PHEMA hydrogels offer oxygen permeability, although lower compared to silicone hydrogels. PHEMA contact lenses have been explored as drug-eluting bandages with release durations from hours to days due to their open network [33, 34]. Strategies like nanoparticle doping, molecular imprinting, or surface coatings can extend drug release. Fabrication of PHEMA devices involves polymerizing HEMA monomers in a mold with a crosslinker like ethylene glycol dimethacrylate [31]. Cast-molding is used for contact lenses, which are then hydrated. PHEMA can also be electrospun in certain solvents to form fibrous scaffolds for ocular cell culture. As an acrylate polymer, PHEMA exhibits good optical transparency and is foldable.

### 2.2. Biodegradable materials

Biodegradable polymers are designed to gradually break down in physiological conditions. Implants made from these polymers do not need to be removed after the complete release of their drugs. The degraded products must be biocompatible so they can be metabolized or excreted safely. The most widely used biodegradable polymers for ophthalmic devices are the aliphatic polyesters: poly(lactic-co-glycolic acid) (PLGA) and its homopolymers polylactic acid (PLA) and polyglycolic acid, as well as poly(caprolactone) (PCL). All four polymers are used in FDA-approved devices. Here, we discuss these material platforms with an emphasis on their fabrication and properties.

#### 2.2.1. Poly(lactic-co-glycolic acid) (PLGA)

PLGA has been the workhorse biodegradable polymer in FDA-approved ocular implants for sustained drug delivery [15]. It is a copolymer of lactic acid and glycolic acid. By adjusting the ratio of these monomers (e.g. 50:50–75:25) and their molecular weights, the degradation rate can be tuned from a few weeks to months [35]. PLGA is an amorphous, hydrophobic polyester and is relatively brittle in its solid form (e.g. a 50:50 PLGA film has a Young’s modulus on the order of 1–2 GPa) [36]. As it absorbs water, it softens and degrades; its matrix becomes more porous and pliable until it fragments and releases lactic and glycolic acids, which are subsequently eliminated. Despite PLGA’s biocompatibility, these byproducts can lower local pH, causing irritation or inflammation. The degradation of PLGA devices typically occurs within 6 months for a single ocular implant, as beyond that the acidic buildup can become problematic, and the structural integrity can be diminished [37]. For example, the Ozurdex^®^ intravitreal implant for macular edema is made of PLGA (with a 65:35 lactic:glycolic ratio) and releases dexamethasone over ~3–6 months, after which it fully biodegrades [38]. PLGA is commonly processed via solvent-based techniques. It can be dissolved in volatile solvents (e.g. dichloromethane), mixed with the drug, and then either casted, spray dried, or extruded into the solution. Microsphere fabrication is also used, in which PLGA and the organic phase of the drug are emulsified in water to form microspheres that harden as the solvent evaporates [39, 40]. Given its *T*_g_ of 40 °C–60 °C, PLGA is also used in heating-based manufacturing.

The erosion kinetics of PLGA-based intraocular systems typically occur over a timescale of weeks to months. A rabbit intravitreal study evaluating a bioerodible PLGA-based dexamethasone implant demonstrated erosion over approximately 4 weeks, with complete disappearance by week 6 [41]. These findings were corroborated by a related rabbit study, which reported near-complete erosion by approximately 1.5 months, with minimal residual mass observed by week 6 [42].

However, it is important to recognize that intraocular biodegradation does not occur without consequence in all contexts, particularly within the anterior segment. The intracameral biodegradable bimatoprost implant (DURYSTA^®^), composed of a biodegradable polymer matrix, provides a clinically relevant example. The FDA-approved labeling for this product includes warnings regarding corneal adverse reactions and an increased risk of corneal endothelial cell loss, likely related to implant breakdown and fragment-endothelium interaction, explicitly restricting use to a single implant per eye without retreatment. Analysis from the FDA clinical review revealed that ⩾20% corneal endothelial cell density loss occurred from Cycle 2 onward in treated eyes, with a dose-dependent increase in frequency, underscoring the cumulative endothelial risk associated with repeat intracameral administrations [43]. Similarly, the FDA-approved Ozurdex^®^ utilizing a PLGA-based biodegradable polymer system is contraindicated for use in the anterior chamber, which can lead to corneal decompensation.

#### 2.2.2. PLA

PLA is a polymer of lactic acid that exists in two enantiomeric mixes. In ophthalmic applications, poly(L-lactic acid) (PLLA) and poly(D, L-lactic acid) (PDLLA) have been used. PLLA is semi-crystalline with a melting point around 170 °C, and a Young’s modulus of 2–4 GPa in its initial form [44–46]. PDLLA is amorphous and less stiff and brittle. PLA is hydrophobic and has higher mechanical strength than PLGA. However, it lacks glycolic units that generally result in slower degradation, often between 6 months to 2 years. Degradation yields lactic acid, which can be metabolized in the body. PLA can also be processed similarly to PLGA. For example, bioabsorbable sutures (e.g. Vicryl^®^) contain PLA and are melt-spun or extruded. However, for drug matrices, melt processing at ~170 °C is high for most drugs; instead, solvent casting or extrusion at lower temperatures is typically used. Electrospinning PLA is common for tissue engineering. PLA nanofiber mats have been explored for engineering corneal stroma, offering a temporary scaffold that cells can populate [45, 46].

PLA exhibits a slow, bulk-erosion-dominated mass-loss profile *in vivo*, with reported weight losses of ~10%–20% over several months and ~50%–60% over approximately 12–15 months, depending on polymer chirality, molecular weight, and implantation site [47]. In peri-orbital and other soft-tissue environments, PLA remnants have been observed for up to ~9 months or longer, indicating chronic, multi-month to multi-year degradation kinetics rather than rapid resorption. Within this context, the biological response to PLA is generally mild and strongly governed by fragment size. Across 71 *in vivo* PLLA implant studies, approximately 55% showed mild foreign body reactions characterized by foreign body giant cells (FBGCs) and fibrous capsule formation without clinical symptoms [48]. FBGC formation was minimal when polymeric fragments remained <4 *μ*m, while fragments >40 *μ*m consistently triggered a multinucleated giant cell response. Critically, long-term ocular safety data corroborate these mechanistic insights. Longitudinal data from PLA-based intracameral implants [49] indicate a transient corneal endothelial cell density loss of 1.3% to 3% within the first month, which is attributed to the procedural ‘incision effect.’ Crucially, in chronic 6-to-20 month observations, there was no statistically significant progressive loss attributable to PLA degradation products. corneal endothelial cell density levels remained stable and significantly above the critical physiological threshold of 500 cells mm^−2^ required for corneal transparency [50].

#### 2.2.3. PCL

PCL is a biocompatible and biodegradable polyester that has gained attention for long-term drug delivery. PCL is a semi-crystalline polymer with a low melting point (~60 °C) and a *T*_g_ around −60 °C, making it flexible at body temperature. As a solid, PCL has a Young’s modulus on the order of hundreds of MPa [51]. It can elongate 300%–500% before breaking, with a tensile strength around 10–20 MPa [52]. PCL degradation produces 6-hydroxycaproic acid, which can be metabolized. In ocular studies, PCL implants remain at least partially intact for many months, enabling prolonged drug release [28]. PCL can be melt-processed at relatively low temperatures (~60 °C) which allows manufacturing techniques like 3D printing and simple extrusion, without degrading drugs. A recent study used 3D printing to fabricate PCL implants loaded with triamcinolone acetonide for intraocular use [53]. Studies have shown PCL implants achieving drug release over months to a year or longer. PCL’s hydrophobicity makes it a good carrier for hydrophobic drugs, which partition into its matrix. Even though hydrophilic drugs can be loaded, they often result in crystalline dispersions that may leach out quickly once water infiltrates. While no PCL-based ocular drug implant is on the market yet [28], experimental studies have used PCL (e.g. in subconjunctival implants for sustained drug delivery in [15]). Electrospun PCL scaffolds have been used in corneal tissue to support cell growth [54].

Studies of subconjunctival polymer microfilms have provided additional insights into degradation behavior and biocompatibility. In a rabbit model, PLGA 50:50 and poly(D, L-lactide-co-caprolactone) (PLC 70:30, a PCL-rich copolymer) microfilms were implanted subconjunctivally and monitored over 6 months. PLGA films underwent bulk degradation characterized by increasing water uptake and detectable mass loss beginning around 3 weeks, whereas PLC 70:30 exhibited a slower molecular weight decline with measurable mass loss occurring only after approximately 8 weeks. Clinical examinations and histological assessments throughout the study period confirmed that both formulations were biocompatible and safe for subconjunctival placement, producing only mild peri-implant inflammatory infiltrates while preserving surrounding ocular structures [15, 55].

For slower-eroding polymer systems, PCL-based intravitreal implants have demonstrated prolonged persistence with minimal inflammatory sequelae. In one long-term rabbit study, PCL intravitreal implants sustained drug release for 55 weeks, retaining approximately 79% of the initial drug load at study termination. Clinical and histologic evaluations indicated excellent tolerability of these devices over this extended period [56].

Overall, these *in vivo* data suggest that broad generalizations regarding the harmlessness of chronic polymer erosion require refinement. A more nuanced conclusion is warranted: polymer degradation is generally well tolerated when devices are appropriately engineered and positioned. However, chronic exposure to degradation byproducts or particulates, particularly in the anterior segment and with repeated dosing, can result in clinically significant adverse effects, including corneal endothelial cell loss.

### 2.3. Comparative overview of polymeric materials

To help synthesize these observations, we have compiled a comparative overview of the principal synthetic polymers currently employed in ocular drug delivery. This summary contrasts non-biodegradable platforms (PMMA, PP, silicone elastomers, PVA, PHEMA) with biodegradable polyesters (PLGA, PLA, PCL) across several critical dimensions: degradation behavior, mechanical and optical properties, drug-loading feasibility, processing constraints, representative clinical applications, and primary limitations. By organizing these parameters side by side, table 1 illustrates how effective material selection depends on matching polymer characteristics to the anatomical site, desired treatment duration, and physicochemical properties of the drug, rather than defaulting to a single ‘universal’ solution.

## 3. Fabrication methods for ocular drug delivery implants

### 3.1. Requirements and strategies for ocular implant fabrication

The fabrication of ophthalmic drug delivery systems requires biocompatible materials and precise manufacturing. These systems must maintain optical clarity, comfort, and stability for daily wear. To achieve reliable drug release kinetics and consistent dosing, manufacturing should reach micron- or submicron-scale precision and employ advanced micro- or nanofabrication techniques such as molding, micro-electro-mechanical systems (MEMS), 3D printing, or electrospinning. The fabrication process must accommodate the integration of drug reservoirs and drug-loaded materials and release-control mechanisms while ensuring sterility and long-term stability. Structurally, the fabrication process must be compatible with the unique geometries and mechanical properties demanded by ophthalmic applications, including a curved profile, optical transparency, softness, and high-water content [57]. It must also meet stringent high-resolution requirements to ensure precise functional features, such as responsiveness to multiple physical stimuli and oxygen exchange [58].

#### 3.1.1. Fabrication strategies

Fabrication of drug delivery systems falls into two categories, each with distinct designs: drug-incorporated implants and cavity-based systems. In drug-incorporated implants, the therapeutic is either directly mixed with the implant materials or coated onto the device. Cavity-based systems function as reservoirs to contain the drug. Beyond the drug-containing cavity, ancillary components must be carefully engineered. These include the release mechanism (e.g. simple diffusion or stimulus-triggered release), the release profile (e.g. release rate and sequence of drugs), and the channels which deliver the drug to the target site [59]. Although not all systems require these features, proper fabrication is crucial when they are required. Moreover, fabrication methods should not alter the functions or properties of a drug.

#### 3.1.2. Drug loading strategies

Drug loading methods and timing vary depending on the drugs, implant materials, fabrication techniques, release mechanisms, and device structures. Matrix-type devices employ a pre-loading strategy to incorporate the drug into the material before or during fabrication. This can be done by mixing the drug with the polymer or by leveraging specific molecular interactions, such as covalent bonding or electrostatic binding, to the polymer matrix [60, 61]. Post loading strategies for matrix-type devices involve soaking the device in a concentrated drug solution to allow diffusion into the matrix, forming a drug coated layer on the device surface, or using a co-axial nozzle to coat extruded struts with drugs. For reservoir-based devices, the drug is typically introduced into an internal cavity by microinjection, requiring aseptic handling, sterilization compatibility, and reliable resealing of the injection port [59]. The choice of pre-loading or post-loading is dictated by the drug’s compatibility with the fabrication steps. Some drugs cannot survive processes involving elevated temperatures, UV curing, high voltage, organic solvents, or long vacuum steps. Such drugs should be loaded after fabrication, whereas more stable compounds can be embedded during fabrication to achieve higher drug loading and longer-term release [62].

There are a variety of methods and strategies that can be applied to fabricating ophthalmic drug delivery systems. Here, we discuss mold template methods, 3D printing, MEMS, and electrospinning (figure 4 and table 2).

### 3.2. Mold templates

Template-based methods serve as a foundational reverse mold for shaping micro- and nano-scale ophthalmic devices. They are widely applicable and low-cost as molds can be reused and are compatible with multiple materials, scales, structures, and sterilization processes. When combined with other fabrication methods such as injection, spin coating, and dip coating, mold templates enable the creation of drug-loaded bulk materials and cavity-based reservoirs.

#### 3.2.1. Molding and injection molding

Solid structures and drug-loaded materials can be fabricated by injecting or depositing precursors into a defined mold. This is especially common for structures with simple, well-defined curvatures [71]. Molds are normally designed to be two separable parts that assemble to form the cavity. Depending on the materials of the mold and the precursor, a specific releasing layer, such as a dichloromethylsilane layer, may be needed [72].

Multiple molding and injection molding steps are critical for high-resolution fabrication. High-resolution hydrogels or rubber master molds can be fabricated using silicon-wafer photolithography or 3D printing. With multiple mold processes, features on the order of tens of microns can be realized [73]. Proper releasing materials can also be chosen if a fabricated mold does not fit the device’s material. Although injection molding is a mature technology, one must remove imperfections such as bubbles to avoid defects.

#### 3.2.2. Spin-coating molding

During spin-coating molding, liquid precursors are dispensed into a high-fidelity mold and spread into a conformal film by rotation [74]. Immediately after dispensing, a brief vacuum hold or gentle sonication removes trapped air and flattens the meniscus. Film thickness and uniformity are set by spin-speed, time, and fluid viscosity. Higher speeds and longer spins yield thinner, more uniform walls. For hollow geometries, such as drug reservoirs, centrifuging with controlled drying or partial curing allows the material to preferentially wet and solidify on the mold’s inner surface, leaving a central cavity. A final curing step maintains the part geometry, after which the structure can be released.

This approach also supports sequential deposition for multi-layer drug reservoirs. An initial spin forms a fast-dissolving or high-diffusivity inner layer. After a partial cure, a second spin applies a slower-release outer layer. Layer interfaces are defined by cure timing and surface treatment (e.g. plasma) to promote adhesion and limit intermixing. Than *et al* used this method to fabricate multilayer microneedles with different release kinetics. In their process, a small volume of methacrylated hyaluronic acid (MeHA) aqueous solution is applied into a PDMS female microneedle mold pre-treated by plasma for improved wettability, then centrifuged into cavities. After overnight ambient drying, MeHA preferentially coats the cavity walls, forming a hollow outer shell. The remaining volume is filled with hyaluronic acid (HA) solution and air-dried to create a solid HA core. A pure HA layer is then cast over the mold to form the flexible backing, and finally, the patch is peeled off, followed by UV curing [75]. Kim *et al* used this method to construct a device with a C-shaped main reservoir and its end-features—-a refill valve on one side and a controlled-release filter seat on the other—-in a single molding step. Functional components are placed into the mold with precursors and permanently captured during curing, eliminating secondary alignment fixtures and saturable add-ons [76].

#### 3.2.3. Dip-coating molding

In dip-coating molding, a clean, surface-conditioned mold insert is immersed in a degassed liquid precursor and withdrawn at a controlled speed to form a uniform wet film. A short leveling dwell (and optional slow rotation) reduces edge beading, after which the wall is fixed by curing. Thickness is set by withdrawal speed, viscosity, and solvent volatility. Multiple dips with partial cures can build precise multilayers. Hollow geometries can be formed by coating over a sacrificial core such as PVA or sugar.

Dip-coating molding is simple and low-cost, with broad material compatibility and fine thickness control via withdrawal speed and viscosity [77, 78]. It enables multilayers and conformal coatings on curved microscale parts and can form seamless hollow reservoirs using sacrificial cores [79, 80]. However, dip coating has disadvantages. Gravity-driven drainage can cause edge beading, solvent-evaporation defects, nonuniform curing in shaded and deep regions, layer intermixing without partial cures, and demolding issues on high-aspect features [81, 82]. Most problems can be mitigated by brief leveling dwells or slow rotation, rheology modifiers, controlled drying, partial cures with surface activation, transparent molds or dual-sided exposure, and release agents or sacrificial cores [83, 84].

Excluding integration with other fabricating methods, molds can be reusable or single-use. Reusable mold methods can be applied to materials if the fabricated material and structure can be released without damaging the mold, like a hydrogel sheet from an elastomer mold or an elastomer microneedle from another elastomer mold. Single-use ‘sacrificial’ molds are temporary structures made of materials like photoresist or metals that are removed by dissolution or etching. Sacrificial methods enable hollow microstructure formation for complex, drug-loaded constructions, while further processing can be done after removal [85]. These strategies are commonly used to release materials that adhere well to the substrate [86]. Generally, it is better to use cost-effective materials for sacrificial molds.

Molding is a widely used, low-cost, and straightforward fabrication method that scales well for simple ocular drug-delivery parts. It works well with repeatable shapes, smooth surfaces, and high throughput with commodity polymers and silicones. It is less effective with very fine or highly complex internal features, such as tortuous microchannels and sub-100 *μ*m details [87–89]. Demolding can introduce defects, seams, or flash, and controlling wall thickness can be challenging. Cavity parts fabricated using centrifuge-assisted or dip-coated molding methods often show cross-sectional non-uniformity. Additional drawbacks include trapped bubbles and voids, polymer shrinkage, and limited multi-material integration.

### 3.3. 3D printing

3D printing techniques, including light-assisted (e.g. stereolithography (SLA) and digital light processing (DLP)) and extrusion-based methods (e.g. fused deposition modeling (FDM)), are increasingly used for ophthalmic drug delivery applications [90, 91]. Compared with other fabrication methods, 3D printing is fast and customizable. It can produce high fidelity 3D constructs using computer-aided design and manufacturing with minimal material waste.

Mohamdeen *et al* developed a method combining hot-melt extrusion (HME) with FDM to produce filaments of ethylene-vinyl acetate (EVA)/ PLA/ timolol maleate at a ratio of 84:15:1 (wt/wt) at 10 V using a 2.5 mm circular die [92]. Different winding speeds generated filaments with a desired diameter ranging from 2.6 to 2.9 mm, achieving good printability, mechanical strength, and surface quality suitable for creating contact lenses. Despite successful fabrication, the printed drug-eluting lenses exhibited a rapid burst release followed by only three days of sustained drug release of glaucoma medication timolol. SLA and DLP leverage photopolymerization to build high-resolution, biocompatible structures, which are ideal for fabricating drug-eluting contact lenses with optical clarity and precise curvatures. Goto *et al* employed a print resin composed of poly(ethylene glycol) diacrylate (PEGDA, the cross-linker), poly(ethylene glycol) 400 (PEG 400, solvent), diphenyl (2,4,6-trimethyl benzoyl) phosphine oxide (TPO, photoinitiator), and azithromycin (AZI) using vat photopolymerization (VPP), which provides higher resolution than a typical FDM printer. Parameters applied for the printing include a thickness of 50 *μ*m and a curing time of 7.5 s for each layer. Lenses with lower PEGDA concentrations were thicker, and their mechanical strength increased following a second curing step by VPP. Furthermore, the second curing process slowed drug release, while the printed lenses demonstrated effective antimicrobial activity against both gram-positive and gram-negative bacteria *in vitro* [93].

3D printing is a good choice for personalized designs. Xu *et al* used DLP to make punctal plugs with various sizes, shapes, and doses of dexamethasone, a widely used corticosteroid in corneal disease treatment with anti-inflammatory properties [68]. The printed plugs showed good biocompatibility during BalB/3T3 cell cultivation. The drug release profile can also be tuned with different printing recipes. The time required for complete drug release can be controlled within a range of 4 to over 21 d, with a corresponding burst release value of 16.1–17.6 *μ*g ml^−1^ to 4.0 ± 1.3 *μ*g ml^−1^ on the first day.

3D printing offers clear advantages for ocular drug-delivery devices, most notably patient-specific geometries (curvature, fit, reservoir volume), rapid prototyping, and the ability to integrate complex internal channels and release features in a single build. It also presents challenges for translation. Throughput and lot-to-lot consistency limit scalable manufacturing; many platforms struggle with sub-100 *μ*m resolution and surface roughness that can affect comfort and flow. Truly ocular-safe, low-extractables materials are limited, and sterilization (steam, gamma, e-beam, EtO) can deform or contaminate prints [94, 95]. Drug loading and stability may be compromised by heat, UV exposure, and the solvents used in printing. Customization complicates validation and regulatory pathways. Risks can be mitigated with medical-grade materials, encapsulation coatings, controlled post-processing, and reverification of release profiles after sterilization [96, 97]. Ultimately, recipe modification remains crucial, such as tuning viscosity for FDM with different nozzles, different light absorbance of different wavelengths for digital light, and many other parameters. For these reasons, 3D printing has many potential applications for ophthalmic drug delivery.

### 3.4. MEMS fabrication

At the micro- and nanoscale, MEMS fabrication plays a pivotal role in constructing ocular devices such as microneedles and micro-patterned films. Here, the fabrication of patterning like lithography, film deposition like casting and spin coating, chemical vapor deposition (CVD), and physical vapor deposition (PVD), etching like dry and wet etching, and sacrificial methods, are discussed.

Film deposition methods such as casting, spin coating, CVD, and PVD are necessary in device fabrication [98]. They are key to forming thin films for packaging or metal layers. Lithography is a core microfabrication technique in developing MEMS-based ophthalmic drug delivery systems, enabling the precise definition of microscale patterns that dictate the geometry and function of drug reservoirs, channels, and release apertures. For ophthalmic applications, lithography offers the highest resolution and repeatability necessary to form fluidic pathways and microchambers that control drug release rates with submicron precision. This precision is critical for tailoring dose delivery to sensitive ocular environments, ensuring therapeutic efficacy while minimizing side effects. Advanced variations, such as deep UV lithography or soft lithography, can be employed to fabricate high-aspect-ratio features or replicate patterns in biocompatible materials like PDMS, making lithography an indispensable tool for integrating complex, miniaturized drug delivery functionalities into implantable ocular devices [69].

In MEMS fabrication for ophthalmic drug delivery systems, etching methods—both wet (e.g. KOH for anisotropic etching of silicon) and dry (e.g. reactive ion or plasma etching for isotropic or anisotropic removal of material)—are used to sculpt high-precision features in device substrates, such as creating openings for drugs to flow out of. These etching processes are typically integrated with photolithographic patterning as a pre-process to define the etching pattern. In dry etching, plasma or ionized gas species remove material with high aspect ratio control and minimal undercut, making it useful for fabricating through-holes or well-defined cavity walls. Wet etching with alkaline solutions like KOH can rapidly remove bulk silicon in crystallographically controlled planes, which is ideal for forming sloped profiles that aid fluid transport [57].

MEMS fabrication enables micron-scale reservoirs, valves, channels with tight tolerances, multilayer integration, and batch scalability, all of which are difficult to achieve with conventional methods. However, because many MEMS steps use reactive gases, photoresists, etchants, and organic solvents that are not inherently biocompatible, ocular devices require encapsulation, rigorous residue removal, and validation of sterilization compatibility for both materials and payload. When executed with this discipline, MEMS offers precise, compact, and reliable ocular delivery platforms ready for translation [99, 100].

### 3.5. Electrospinning

Electrospinning applies a high voltage to a polymer solution or melt of one polarity, which is then drawn to a collector of the opposite polarity. As the solution or melt is charged, a Taylor cone forms at the tip of the nozzle. Once the electrostatic forces created by the high voltage overcome the surface tension, a jet of the solution is ejected. As the jet travels toward the collector, it may evaporate in the atmosphere, forming solid fibers.

Electrospinning stands out as a versatile and widely studied technique for creating nanofibrous ophthalmic drug delivery systems. Nanofibers fabricated by electrospinning offer high surface area, porosity, and mechanical flexibility, while structurally resembling the extracellular matrix (ECM). These characteristics help enhance drug retention and tissue compatibility [101]. Consequently, electrospun nanofibrous and microfibrous materials present a promising platform for ocular drug delivery and cell-based therapies because of their tunable morphology [102]. Electrospinning works as a nanofibrous scaffold to offer a simple, versatile, and highly adaptable method for fabricating ocular delivery systems. These systems can provide improved bioavailability and sustained release for small molecules, biologics, and cells compared to traditional ocular drug delivery approaches. However, electrospinning has limitations, like low productivity, production inconsistency, difficulty forming complex shapes, and safety issues. Electrospinning has multiple variable parameters like applied voltage, flow rate, distance between nozzle and collector, solution viscosity, and solution conductivity, making consistency difficult. Based on the outcome of fabricated fibers, further steps are required for complex 2D and 3D shapes. Safe use of electrospinning requires attention during high voltages and volatile polymer solvents [44].

### 3.6. Critical comparison and selection guide for fabrication methods

Ophthalmic drug-delivery device fabrication is constrained more by consistently meeting non-negotiable requirements than by the challenge of making ultrafine, complex features. Key requirements include optical clarity, surface comfort, and biocompatibility with minimal extractables and particulates. Additionally, devices must maintain payload stability through processing and sterilization while ensuring highly reproducible dose–release performance. A practical distinction exists between drug-in-matrix and reservoir-based systems. Matrix loading simplifies sealing and assembly but can increase exposure to heat, UV, or solvents, often resulting in burst release and variability. Reservoirs enable higher loading, refillability, and programmable kinetics via membranes, filters, or valves. However, they shift risk toward leak-tight interfaces, tolerance stack-up, and multilayer bonding integrity.

Accordingly, fabrication route selection should be based on mapping device requirements to process capabilities and their specific defect modes. Molding works best for smooth surfaces and scalable throughput, yet it becomes yield-limiting when internal features are highly complex or sub-100 *μ*m. Seams, trapped bubbles, and wall-thickness gradients can also affect comfort or release reproducibility. 3D printing enables rapid customization and complex structures but is constrained by surface roughness and the need to confirm drug stability after post-curing and sterilization. MEMS processes provide unmatched precision and batch repeatability for micro-apertures and valves, though they demand rigorous residue control, packaging, and validated sterilization workflows. Electrospinning can create drug-loaded nanofiber inserts or coatings that increase ocular residence time through high surface area and steady release, but dose uniformity, handling, and sterilization can be challenging to achieve.

## 4. Site-specific strategies for ocular drug delivery implant design

Ocular implants can be broadly categorized by their implantation sites and target tissues [8]. For example, anterior segment implants are typically placed in the conjunctiva, subconjunctival, intracorneal, and anterior chamber regions. These implants are mainly designed to treat glaucoma, corneal neovascularization, and postoperative inflammation [103, 104]. In contrast, posterior segment implants are inserted into the vitreous cavity or the sclera and primarily treat chronic retinal diseases, including AMD, diabetic retinopathy, and uveitis [105, 106]. This section focuses on the principles and strategies of site-specific designs for ocular implants (figure 5). Detailed information on FDA-approved products as well as those undergoing clinical investigation is provided in sections 5 and 6.

### 4.1. Intravitreal implants

Intravitreal implants are advanced drug delivery systems designed to overcome the limitations of conventional solutions delivered by intravitreal injection, including rapid clearance and frequent administration. After administration, therapeutics are eliminated through retinal blood vessels and anterior aqueous humor outflow, with specific elimination mechanisms depending on their physicochemical properties [9]. Small molecules are primarily eliminated by systemic absorption through retinal blood vessels in the posterior segment. Large biologics (e.g. anti-vascular endothelial growth factor (VEGF) antibodies) are mainly eliminated through anterior aqueous outflow [7]. Generally, the half-life of small molecules is less than 10 h, while that of large biologics is about one week [64, 76]. Intravitreal implants can increase drug exposure (>1 month) by slowly releasing therapeutics from a drug reservoir or a matrix. Furthermore, intravitreal implants provide sustained release of therapeutics directly into the vitreous cavity, where the gel-like vitreous humor slows drug diffusion and clearance. This maintains effective therapeutic concentrations over extended periods and reduces patient burden. FDA-approved products include Ozurdex^®^ (dexamethasone, biodegradable), Susvimo^®^ (Ranibizumab, non-biodegradable), Retisert^®^ (fluocinolone acetonide, non-biodegradable), Iluvien^®^ (fluocinolone acetonide, non-biodegradable), and Yutiq^®^ (fluocinolone acetonide, non-biodegradable). These products have shown efficacy in treating chronic ocular diseases such as AMD, diabetic macular edema (DME), and uveitis [107].

Although intravitreal implants have shown clinical success, they present several limitations. Their clinical value remains highly context-dependent, underscoring the need for indication-specific design and risk-benefit optimization. Implantation procedures can cause endophthalmitis, retinal detachment, and vitreous hemorrhage. Long-term exposure to corticosteroid-based devices often results in increased intraocular pressure, glaucoma, and cataract progression [108]. Maintaining the stability of biologics is another challenge during manufacturing and long-term storage [109]. Non-biodegradable implants pose additional concerns about the long-term retention of foreign materials and the need for surgical removal, while biodegradable systems may generate acidic byproducts that cause local toxicity [110]. High device prices and procedural expenses also limit widespread use [111].

The next generation of intravitreal implants will likely focus on broadening their therapeutic scope to include gene-based or RNA-based therapeutics, supported by advances in polymer chemistry and stabilization technologies. Refillable reservoirs, such as the Port Delivery System for ranibizumab, highlight the shift toward minimally invasive, long-acting platforms that reduce the need for procedures and allow physician-controlled dosing [112]. Combination therapies that deliver biologics alongside small chemical agents may address the multifactorial nature of retinal diseases, while smart polymers and stimuli-responsive systems can offer tailored, on-demand drug release [113]. In parallel, emerging modalities such as gene therapy and encapsulated cell technology, may compete with or complement implantable devices, suggesting a future in which intravitreal implants serve as primary long-term solutions or adjuncts in personalized, multimodal therapy [114].

### 4.2. Subconjunctival implants

The subconjunctival space is a vascularized and permeable site for implant placement, enabling drug diffusion through the sclera toward both the anterior and posterior ocular segments [115]. Subconjunctival implants have been studied for treating anterior segment and ocular surface disorders, including uveitis, scleritis, allergic conjunctivitis, peripheral ulcerative keratitis, and glaucoma [104, 116, 117]. When therapeutics are embedded within biodegradable polymeric matrices, subconjunctival implants can achieve sustained, zero-order or near-zero-order release profiles, maintaining consistent therapeutic concentrations for prolonged periods without frequent dosing [103, 118, 119]. Moreover, the versatility in implant design—ranging from micro-reservoir systems to hydrogel systems—-allows precise control of release kinetics tailored to specific diseases. Although no subconjunctival implant has reached clinical use, several efforts have demonstrated promising preclinical outcomes [120]. Biodegradable polymers are typically used to fabricate subconjunctival implants because they facilitate administration and avoid surgical removal [120].

Subconjunctival implants have attracted attention as a less invasive alternative to intravitreal delivery, yet several limitations persist. Although they avoid direct intraocular penetration, surgical implantation beneath the conjunctiva still carries procedural risks, such as subconjunctival hemorrhage, fibrosis, foreign body sensation, and implant extrusion or migration [121]. From a pharmaceutical perspective, common observations in these studies include an initial burst release and a relatively short release duration (~1 month), which can lead to toxicity or low efficacy. In addition, drug bioavailability within the vitreous humor is often limited by rapid clearance through conjunctival lymphatics and the episcleral vasculature, resulting in lower intraocular drug concentrations compared to intravitreal systems [122]. Moreover, creating effective drug delivery pathways for the posterior segment remains a major challenge, as transscleral diffusion is influenced by molecular size, charge, and scleral thickness. This often necessitates high drug loading or permeation enhancers [123].

Future subconjunctival implants are expected to leverage advances in biomaterials and drug-stabilizing strategies to improve delivery to the posterior segment. Currently, novel biodegradable polymers are used to fabricate hydrogels and nanoparticle-incorporated depots. These systems aim to enhance scleral permeation and maintain therapeutic levels of biologics [110]. For example, engineering implants with mucoadhesive or sclera-interacting biomaterials may increase retention and reduce clearance. Stimuli-responsive systems (pH, enzyme, and ultrasound-triggered release) offer a platform for controlled drug release [113]. Refillable or injectable *in situ*-forming depots represent promising strategies as they could minimize surgical procedures and increase patient convenience. From a long-term perspective, combining subconjunctival implants with non-invasive technologies like iontophoresis may act as a multimodal strategy for posterior segment therapy [124].

Subconjunctival implants provide a less invasive pathway for sustained drug delivery when compared with intravitreal systems. Their location outside the ocular globe reduces the risks associated with direct intraocular injection, rendering them an attractive option for chronic anterior segment diseases. While subconjunctival implants may not yet match intravitreal systems for posterior segment targeting, continued material and device innovation could establish them as a clinically valuable, less invasive alternative in selected indications.

### 4.3. Intracameral implants

Intracameral injection refers to the direct administration of therapeutic agents into the anterior chamber of the eye. This approach has been increasingly used to achieve highly localized drug delivery. Typically, intracamerally injected therapeutics aim to treat endophthalmitis prophylaxis, inflammation in the anterior chamber, and glaucoma [125, 126]. Intracameral implants represent an advanced drug delivery approach specifically designed to overcome the anatomical and physiological barriers of the eye [9]. These implants are inserted directly into the anterior chamber and may be anchored to the trabecular meshwork, enabling release at the site of action [127]. By bypassing the corneal epithelial layers and tear film, intracameral implants achieve significantly higher bioavailability at the target site, resulting in greater therapeutic efficacy [128]. FDA-approved intracameral implants include DURYSTA^®^ (bimatoprost; biodegradable) and iDose^®^ TR (travoprost; non-biodegradable) [129, 130].

Intracameral implants are effective for postoperative inflammation and glaucoma but face several challenges. Intracameral implantation can induce hyphemia, corneal endothelial damage, and angle obstruction [131]. Biodegradable implants can provoke local inflammation and inconsistent release as degradation accelerates. Non-biodegradable implants raise concerns about foreign-body retention and the need for removal [132]. Long-term endothelial safety remains a major limiting factor for widespread use.

Future intracameral implants are expected to focus on enhancing safety profiles, broadening drug diversity, and controlling release kinetics. Advances in biocompatible polymers and smart hydrogel systems may allow for more consistent release kinetics while minimizing burst effects and inflammatory byproducts. Combination therapy, such as co-delivery of anti-inflammatory and antifibrotic agents, may prevent postoperative complications more effectively. Incorporating biologics, RNA therapeutics, or gene-editing tools could expand the scope of intracameral systems beyond current therapeutics.

In comparison with topical or subconjunctival delivery, intracameral implants provide superior target-specific bioavailability and prolonged therapeutic exposure in the anterior chamber. However, because they involve direct anterior chamber manipulation, they carry higher procedural risk and require stringent safety requirements for the corneal endothelium. Consequently, the clinical utility of intracameral implants depends on the meticulous balancing of efficacy, safety, and device permanence, underlining the need for disease-specific implant designs.

### 4.4. Intracapsular implants

Intracapsular implants refer to medical devices or drug delivery systems placed within the capsular bag of the crystalline lens. These implants are commonly injected together with an artificial IOL after cataract surgery. The capsular bag protects implants from exposure to intravitreal environments, increasing stability and safety [133]. Traditional intracapsular devices are limited to IOLs for visual rehabilitation; however, recent innovations have expanded their role to therapeutic drug delivery [134]. Implanting a drug-loaded IOL within the capsular bag offers several advantages: mechanical stability due to zonular support, reduced risk of corneal endothelial damage compared to anterior chamber placement, and minimal interference with aqueous humor outflow [133, 135]. Limited interactions with external environments allow intracapsular reservoirs to provide sustained release profiles over extended periods. Drug reservoirs are integrated into IOL appendages (i.e. haptics) that hold the lens optic in place within the capsular bag. Thus, the optical properties of the central lens remain uncompromised, preserving visual quality. From a pharmacological perspective, the stable, localized position of an intracapsular implantation can delay release and increase residence time of therapeutic agents. Intracapsular implant systems can deliver anti-inflammatory agents to mitigate postoperative inflammation, antibiotics to prevent infectious complications, and antifibrotic agents to inhibit aberrant lens epithelial cell proliferation and posterior capsule opacification. By maintaining therapeutic concentrations directly in the intraocular microenvironment, intracapsular implants reduce reliance on patient adherence with topical regimens [134, 136].

The application of intracapsular implants presents several challenges. Relative to other ocular implant platforms, intracapsular systems are best suited for postoperative cataract surgery, highlighting a trade-off between anatomical safety and therapeutic flexibility. Difficulty controlling release kinetics is another issue. The capsular bag can act as a barrier for drug release while also protecting the implants. Refilling therapeutics can be challenging since the IOL is permanently implanted. There is potential for an initial burst release followed by subtherapeutic levels, depending on polymer degradation and drug–lens interactions. Moreover, drug reservoirs can reduce optical clarity, mechanical stability, and biocompatibility, which can raise concerns about posterior capsule opacification, foreign body response, and long-term safety [137]. Clinical translation has also been hampered by the limited range of therapeutics (mainly antibiotics and corticosteroids) and the lack of large-scale, long-term safety studies.

Advances in coating chemistry, nanostructured reservoirs, and stimuli-responsive materials should enable more consistent, prolonged drug release without sacrificing optical properties. Developing refillable systems could significantly increase clinical feasibility [131]. Current studies are confined to single drug deliveries rather than combinations of multiple drugs. Although controlling the release rate of each drug would be challenging, it could improve therapeutic efficacy. The integration of biologics and gene-based therapeutics within IOL platforms remains a promising frontier, provided that stability and controlled-release challenges can be addressed.

## 5. Clinically available ocular therapeutic implants: success and failures

Implantable ocular therapeutic systems have been developed and approved by the FDA for the management of intraocular inflammation, retinal disease, the postoperative state, and glaucoma (figure 6). In the field of retina, this is a way to avoid frequent intravitreal injections for patients. In the field of glaucoma, it holds promise for improving patient adherence to medication and reducing the need for eye drops. For the postoperative state, it reduces the burden of eye drops after surgery. In some fields, the ability to provide continuous drug delivery of small peptides enables therapies for previously untreatable diseases, such as macular telangiectasia type 2 [138]. For clinical trials, the regulatory paths require a confirmation of effectiveness compared to current treatment, along with an acceptable safety profile [139]. These devices require an excellent safety profile to match current therapies. We will review implantable ocular therapeutic systems by disease category (figure 7), discussing devices with FDA approval, devices that are actively in the research process, devices in clinical trials, and devices that failed approval (table 3).

### 5.1. Retinal disease

#### 5.1.1. Anti-VEGF drug delivery

Anti-VEGF therapy is a mainstay treatment for many retinal diseases including neovascular AMD, diabetic retinopathy, DME, central/retinal vein occlusions, and choroidal neovascularization, myopic choroidal neovascularization, and retinopathy of prematurity. Pegaptanib, an RNA aptamer that binds VEGF165, was the first intravitreal FDA-approved anti-VEGF agent in 2004. It was followed by multiple other anti-VEGF medications, all of which are limited by the need for periodic repeated injections [140].

To further extend drug delivery of anti-VEGF therapies, the Port Delivery System (Genentech) was approved for neovascular AMD in 2021, DME in 2025, and diabetic retinopathy in 2025. This device is an intraocular drug reservoir that is a transscleral implant placed in the pars plana of the eye, providing controlled release of ranibizumab. A septum to refill the device is accessible under the conjunctiva. The 20 *μ*l drug reservoir is filled with concentrated ranibizumab, which is released into the eye via diffusion through a release control element. The Port Delivery System is constructed of a polysulfone body, silicone septum, and titanium release control element. Pharmacokinetic studies from the Phase 2 study demonstrated that intraocular ranibizumab concentrations were within the range expected for monthly ranibizumab injections for a minimum period of 9 months and were above the limits of measurement for 16 months [141]. Refills are every 24 weeks for neovascular AMD and DME and every 36 weeks for diabetic retinopathy [142, 143]. The device underwent a voluntary recall in 2022 after reports of septum dislodgement into the body of the implant. A root cause analysis found insufficient bonding between the septum and overmold, and excessive septum deformation caused by the refill needle insertion.

Component-level changes were implemented to double septum-overmold retention force and to reduce the force required to insert the needle by half [144]. The contact area between the septum and overmold was increased, less epoxy was used to bond the septum and implant body to avoid excess epoxy exposure, and a second oven treatment cycle was implemented to vaporize uncured epoxy. The refill needle was updated to include a small amount of silicone-based lubricant to the exposed surfaces of the inner and outer cannula. The lubricant is immobilized on the refill-needle cannula surface through a partial crosslinking process, specifically designed to minimize the risk of silicone migration. Future work on the implant includes the possibility of incorporating different pharmaceuticals to treat other diseases. The Burgundy trial is currently evaluating the port delivery system with Zifibancimig, a novel VEGF and Ang2 DutaFab [145].

The Posterior MicroPump Drug Delivery System (Replenish, Inc) was evaluated with a first-in-human safety and surgical feasibility study in 2014 for DME. The device uses a transscleral implantation, with the pump placed under the conjunctiva and Tenon’s capsule and a transscleral tube entering the posterior chamber. It is fabricated using MEMS processing. The Posterior MicroPump Drug Delivery System consists of hermetically sealed electronics for power and control of drug delivery. It features an electrolysis pump, a 60 *μ*l drug reservoir chamber, a one-way check valve that opens when the internal reservoir pressure exceeds cracking pressure, a refill port that is accessed transconjunctivally, and an intraocular cannula that is implanted in the pars plana, which allows a microdose of drug into the vitreous cavity. The electrolysis chamber is used to deliver the desired dose volumes by generating H_2_ and O_2_ gas to increase pressure in the chamber and force the drug through a cannula into the eye. These gases recombine in the chamber to form water once the pump is turned off [146]. In the Phase 1 trial, a pre-filled device was implanted into patients with DME for a period of 90 d and then explanted. Immediately after implantation, the pump was wirelessly connected to a telemetry system, which received pump information and gave a command for a single drug delivery. During the follow-up period, the pump remained but was not programmed to deliver further doses of ranibizumab. The device delivered the target dose for 7 of the 11 patients implanted in the allotted time, while a reduced dose was delivered in the remaining 4 patients. In the reduced dose subgroup, the micropump had a slower rate of delivery; infusion was subsequently stopped, and the patient received an intravitreal injection. Bench evaluation attributed it to damage to the device during implantation [147].

The Port Delivery System has been able to demonstrate long-term delivery of anti-VEGF fab fragments and is under investigation to be used with other classes of medications such as DutaFabs [145]. In this role, it could provide a platform for broadly treating retinal diseases. However, this device cannot dynamically change drug delivery levels for the patient’s individual needs and does not deliver pharmaceuticals on demand. The subconjunctival location of the device, which extends through the sclera, has been associated with elevated rates of conjunctival erosion, exposure, endophthalmitis, and vitreous hemorrhage [148, 149]. In contrast, the Posterior MicroPump Drug Delivery System is more complex, requiring electrical power to function and telemetry for communication. This allows it to provide customized levels of drug tailored to patient needs. As it was studied in a limited 11 patient Phase 1 trial, surgical complication rates and long-term device risks are currently unknown. Compared with other intravitreal or intracameral injections, implantation procedures carry the risks of surgery, surgical complications, postoperative surgical complications, and the risks of anesthesia [149]. They also require recovery after the procedure, with decreased visual acuity commonly observed in the postoperative period [150].

#### 5.1.2. Steroid and antiviral delivery

The Duraset technology by EyePoint Pharmaceuticals is an injectable drug delivery system for small molecules that can deliver drugs over three years with a single injection [151, 152]. It allows miniaturized drug delivery through 27-gauge needles [153]. Products used with it can be bioerodible or non-bioerodible. The Duraset technology provides extended drug delivery of ganciclovir with Vitrasert^®^, steroids with Retisert^®^, Iluvien^®^, and Yutiq^®^, and an EYP-1901, a tyrosine kinase inhibitor (TKI) [154].

Vitrasert^®^ (Chiron Corporation/Bausch and Lomb/pSivida Ltd) was approved in 1996 to treat cytomegalo-virus retinitis [155]. It is implanted into the vitreous chamber via a trans-scleral incision and sutures anchoring it to the pars plana. It releases 1 *μ*g h^−1^ of ganciclovir for a 4–5 month period [156]. A 4.5 mg ganciclovir and 0.25% magnesium stearate pellet is coated with 10% PVA and dried. Then, it is coated with ethylene vinyl acetate except for its top surface and coated again with 10% PVA. The nonpermeable ethylene vinyl acetate reduces the surface area of diffusion, while the PVA surface allows diffusion. Ganciclovir is dissolved as water enters the device, resulting in zero-order kinetics [157]. A suturing strut is attached, and the device is gas sterilized [158, 159].

A similar device, the fluocinolone intravitreal implant Retisert^®^ (Bausch and Lomb), was approved for the treatment of chronic non-infectious uveitis affecting the posterior segment of the eye in 2005 [160, 161]. The Phase 3 pivotal trial demonstrated that patients treated with Retisert^®^ had a lower recurrence rate of uveitis compared with sham control subjects. The Retisert^®^ device is implanted into the vitreous chamber by being sutured to a 3 mm wide pars plana incision. The device is 3 mm × 2 mm × 5 mm and has a 0.59 mg fluocinolone acetonide drug core, surrounded by a silicone cup with an orifice that allows drug release. A PVA membrane is placed between the fluocinolone acetonide drug core and the release orifice. The silicone cup is attached to the PVA suture strut with silicone adhesive. Steady state release rates start at approximately 0.3–0.4 *μ*g d^−1^ for 30 months. Bausch and Lomb sent a letter to customers stating that Retisert^®^ will be discontinued in 2026 due to manufacturing difficulties [162].

Iluvien^®^ and Yutiq^®^ (ANI Pharmaceuticals) are intravitreal non-bioerodible fluocinolone acetonide releasing implants that were respectively FDA-approved for DME [163] in 2014 and non-infectious uveitis [160] in 2018. They contain 0.18–0.19 mg of fluocinolone acetonide which is released over a period of 3 years (0.25 *μ*g d^−1^) [164]. The device consists of an internal matrix containing fluocinolone acetonide, surrounded by a non-bioerodible polyimide tube, with a non-permeable cap on one side and a permeable PVA cap on the other side. The cylindrical device is 0.37 mm in diameter, 3.5 mm long, and is injected into the vitreous cavity with a 25-gauge inserter.

Ozurdex^®^ (Allergan Pharmaceuticals) is a biodegradable dexamethasone releasing intravitreal implant with FDA approval for management of non-infectious uveitis, and macular edema due to diabetes, branch, or central retinal vein occlusion [165]. It is 0.46 mm in diameter, 6 mm long, and is injected using a proprietary 22-gauge injector. 0.7 mg of micronized dexamethasone is released over a period of 3–6 months (0.6 *μ*g d^−1^) through the dissolution of a PLGA matrix. A HME process is used to fabricate the device, which is ideal as dexamethasone is relatively heat-stable. A 60:40 ratio of drug and polymer is mixed between rotating screws above the *T*_g_. The melt is extruded through a die to form a filament which is cut to length [166]. There is limited intermolecular interaction between the micronized dexamethasone and the PLGA, preventing dexamethasone from diffusing through the polymer matrix. This results in a two-phase system of dexamethasone crystals embedded in a PLGA matrix without dissolving [167].

I-vation^™^ (Surmodics) is a titanium helical device with a cap that is implanted in the pars plana through a 0.5 mm sclerotomy with a cap that sits subconjunctival [168]. It releases triamcinolone acetonide over 24 months. The device is 0.4 mm long by 0.2 mm wide. The titanium device is coated with 925 *μ*g of triamcinolone acetonide formulated with the non-degradable polymers poly(methylmethacrylate) and ethylene vinyl acetate using proprietary coating processes developed by SurModics, Inc [169, 170]. The device features a helical design that increases overall surface area. Side effects included cataracts in 17 of the 18 subjects in the Phase 1 trial [171]. Phase 2 was terminated [168].

Steroids, such as dexamethasone and triamcinolone, can be injected directly into the eye using a 30-gauge needle. However, direct injection into the eye is limited by dexamethasone’s short half-life of 3.5 d [172] and the burst release kinetics of triamcinolone acetonide [173]. To stabilize steroid drug delivery, there are many commercially available extended drug delivery devices. Retisert^®^ and Iluvien^®^/Yutiq^®^ are both approved for management of non-infectious uveitis, with Retisert^®^ having higher release rates of fluocinolone acetonide but requiring surgical implantation. Iluvien^®^/Yutiq^®^ are inserted with a custom preloaded injector. Likewise, the dexamethasone implant, Ozurdex^®^, is provided with a preloaded injector. Although these have a long history of extended drug delivery to the eye, steroid-based systems are limited to side effects of the steroids themselves. These include cataract formation and an increase in intraocular pressure [168, 171, 174]. Further, the surgically implantable device that was approved for use, Retisert^®^, has recently been discontinued.

#### 5.1.3. Neuroprotection

Neurotech has been refining encapsulated cell therapies for ocular and retinal disease since the early 2000s. NT-501, Revakinagene taroretcel-lwey (Encelto^™^) delivers ciliary neurotrophic factor (CNTF) for the treatment of macular telangiectasia type 2, which was approved by the FDA in 2025 [138]. NT-501 is a cylindrical device 1 mm in diameter and 6 mm long, with a suture clip on one side [175].

It is implanted in the pars plana and held in place with a retention suture. Manufacturing details of the device are proprietary. This device consists of transgenic ARPE-19 cells that continuously produce CNTF, which are in a supportive matrix and surrounded by a semi-permeable membrane. This semi-permeable membrane allows nutrients and oxygen to reach cells while shielding them from immune cells and immune attacks. For macular telangiectasia type 2, the device has been shown to reduce photoreceptor loss (ellipsoid zone area on optical coherence tomography) by 30.6%–54.8% compared to sham over a period of 24 months. This type of technology has shown cell viability for over 14.5 years [176]. NT-501 has also been evaluated for the treatment of geographic atrophy with a Phase 2 clinical trial [177] and retinitis pigmentosa [178, 179]. NT-501 is currently under Phase 2 investigation for glaucoma [175]. Another similar device, NT-503, produced a soluble form of the anti-VEGF receptor (VEGFR) for the management of exudative AMD, although the Phase 2 trial was halted [180, 181].

The Brimonidine Drug Delivery System, BRIMO DDS^®^ (Allergan/AbbVie) is a sustained release brimonidine device made from a biodegradable polymer matrix. Initial studies were loaded with brimonidine tartrate, which was changed to a brimonidine free base to increase active drug loading by 50%. The initial matrix was made of a poly(D, L-lactide) polymer, which was changed to a poly(D, L-lactic-co-glycolic acid)/ (D, L-lactic acid) blend for faster drug release [182]. The BRIMO DDS^®^ is injected into the vitreous cavity for the treatment of geographic atrophy in AMD. In Phase 2 trials, there was decreased GA growth compared to sham by 19%–28% depending on dose, but this was not statistically significant at 12 months [183]. Further evaluation in a Phase 2b trial was terminated early due to slow progression of GA lesions in the enrolled groups [183].

Neuroprotection shows promise for treating multiple diseases, such as glaucoma and macular telangiectasia. However, demonstrating efficacy in a clinical trial setting can be challenging given the slow progression of these diseases. This makes it challenging to demonstrate an improvement in vision between treatment and control groups. To show a demonstrable difference, clinical trials may have to be for multiple years and use advanced imaging or biomarkers of disease progression.

#### 5.1.4. TKIs

EYP-1901, Duravyu^™^ (EyePoint Pharmaceuticals), is a bioerodible implant injected into the vitreous cavity that delivers Vorolanib, a TKI, over a period of 9 months [184]. Drug release is based on a bioerodible version of the Durasert^®^ E sustained release platform. It is under clinical evaluation for wet AMD, DME, and nonproliferative diabetic retinopathy. Patients are being enrolled in an upcoming Phase 3 clinical trial for wet AMD [185], with EYP-1901 provided every 6 months. In DME, it met the primary clinical endpoints at 24 weeks when compared with a single aflibercept injection. The PAVIA trial for moderately severe and severe non-proliferative diabetic retinopathy did not meet the primary endpoint of a 2-step increase in Diabetic Retinopathy Severity Scale at week 36 [186].

Axpaxli, OTX-TKI (Ocular Therapeutix) is a bioresorbable hydrogel that releases 600 *μ*g of Axitinib, a TKI, over a period of 9–12 months. Drug release is based on the ELUTYX drug delivery system. Micronized axitinib is formulated with polyethylene glycol polymer chains that form a hydrogel matrix [187]. This consists of a cross-linked polyethylene glycol polymer chain which slowly releases drugs after insertion through a 25-gauge needle into the vitreous cavity [188]. It has been evaluated for moderately severe and severe non-proliferative diabetic retinopathy and is under current investigation in a Phase 3 clinical trial for wet AMD.

TKIs act on many of the same pathways as anti-VEGF molecules, but in a different location. While anti-VEGF molecules are large protein antibodies which prevent VEGF from binding to the extracellular VEGF receptors, TKIs are small molecules which can act intracellularly [189]. They diffuse into cells and bind to the intracellular domains of receptor tyrosine kinase, providing a broad range of VEGF receptor inhibition. This includes multiple isoforms of VEGF as well as the inhibition of other receptors such as platelet-derived growth factors [189]. Given that they act in a downstream location compared with anti-VEGF molecules, there is significant interest in using them in conjunction with current anti-VEGF therapies to increase treatment durability [189]. Further, while TKIs are small molecules that would normally have a short half-life in the vitreous, they lend themselves to extended drug release compared to many anti-VEGF agents [190]. Since there are no approved TKIs, actual efficacies and uses are still under active investigation.

#### 5.1.5. Integrin based signaling

AXT107 (AsclepiX Therapeutics) is a 20-amino-acid acid collagen IV-derived peptide which self-assembles into an *in-situ* gel depot after intraocular injection [191]. It interacts with integrins α5β1 and αVβ3 to inhibit VEGF and activate TIE2 signaling, promoting vessel stability [192]. It was provided as an intravitreal injection in a Phase 1/2a clinical trial for DME [193]. Dose escalation was performed for 0.1 mg (low dose), 0.25 mg, or 0.5 mg (high dose) [194]. Visual disturbance with an intravitreal injection of a gel is a possibility but was noted to be outside the visual axis in animal models [195]. It also was formulated as a suprachoroidal injection in a Phase 1/2a clinical trial for neovascular AMD [196].

### 5.2. Post-operative state

For the postoperative state, extended drug delivery devices have been used to reduce the need for postoperative drops. This may improve patient compliance and reduce postoperative burden. These implants have been injected into the anterior chamber and placed in the canaliculus [197, 198].

DEXYCU^®^ (EyePoint Pharmaceuticals) is an intracameral dexamethasone steroid spherule injected posterior to the iris at the end of cataract surgery. Dexamethasone release is based on the Verisome technology. Clinical trials demonstrated an increase in the proportion of patients with clearing of anterior chamber cells (inflammation) at day 8 compared with sham injected eyes [197].

Dextenza^®^, an intracanalicular dexamethasone implant, can be used to treat postoperative inflammation and ocular allergies [198, 199]. It is constructed of a biodegradable PEG hydrogel which delivers dexamethasone for 30 d. In a Phase 3 trial after cataract surgery, a greater percentage of patients were pain-free at postoperative day 8 and had 0 anterior chamber cells at day 14 when compared to a placebo implant [198].

MATI Therapeutics has developed a nonbiodegradable L-shaped punctal plug delivery system (Evolute PPDS) which resides in the punctum with a 90° angle that extends into the canaliculus. In a Phase 2 clinical trial, the device compared postoperative nepafenac delivery versus placebo delivery after cataract surgery without routine topical NSAID or steroid, with a primary endpoint of postoperative pain and secondary endpoint of postoperative inflammation. 69% of patients with the implant experienced no pain compared with 38% of the placebo device, and 50% of implanted devices vs 19% of placebo had no inflammation at postoperative day 7 [200]. Critiques of this trial include non-standard postoperative management in the control group without topical steroids or NSAIDs and the possibility of using a non-inferiority trial to reduce patient risk [201].

Standard postoperative treatment for cataract surgery includes a series of eye drops, including topical steroids, antibiotics, and NSAIDs for a period of weeks [202]. However, there has been interest in reducing or removing eye drops after surgery to improve compliance and standardization of postoperative care in a trend known as dropless cataract surgery [203]. Often in dropless cataract surgery, NSAIDs are not used. The use of intracameral antibiotics has called into question the need for further topical antibiotic drops. For example, the 2021 American Academy of Ophthalmology Preferred Practice Pattern for cataract surgery states that while there is evidence that intracameral antibiotics at the time of surgery reduce postoperative endophthalmitis, there is increasing evidence that topically applied antibiotics do not add additional benefit to intracameral antibiotics [203, 204]. Extended drug delivery systems have been designed to reduce the need for postoperative steroid eye drops. These devices are placed at the time of cataract surgery and are either placed intracameral, using a steroid spherule or intracanalicular with a PEG hydrogel that swells after insertion, or using a L-shaped punctal plug system that is under investigation.

### 5.3. Glaucoma

There are multiple devices that provide extended drug delivery for glaucoma. These can be categorized into devices placed into the fornix, subconjunctival space, intracameral, intracapsular-based devices, and contact lens-based devices.

#### 5.3.1. Fornix and contact lens-based delivery

The bimatoprost ring (Allergan/AbbVie) is a flexible circular ocular insert that rests in the fornices of the eye. It consists of bimatoprost mixed into a silicone matrix, with a core PP support. The circular ring is manufactured in sizes between 24 and 29 mm, with sizing determined from the intercanthal distance. Bimatoprost elution goes from approximately 35 *μ*g d^−1^ at day 0–6 *μ*g d^−1^ at day 180, for a release of 2.5 mg over the time period. The device was compared to placebo inserts with timolol topical eye drops twice per day (standard treatment for glaucoma) in a Phase 2 clinical trial. The bimatoprost ring was well tolerated and was non-inferior to timolol for 2 of the 9 time points, with the timolol and sham insert providing approximately 0–1.5 mmHg more intraocular pressure lowering than the bimatoprost insert [205].

The Topical Ophthalmic Drug Delivery Device is a conjunctival insert that has been shown to deliver Latanoprost in beagles [206] and was evaluated for tolerability in healthy volunteers [207]. Other preclinical conjunctival inserts have been developed to deliver timolol, dorzolamide, and other intraocular pressure lowering drugs [208]. The Eye-D (BioLight Life Science) is a subconjunctival insert that delivers Latanoprost. It has been evaluated in Phase 1/2a clinical trials [209].

#### 5.3.2. Canaliculus-based implants and intracameral implants

The canaliculus offers drug delivery from a punctal plug-like device, which can be placed by a physician in the office setting. The MATI Therapeutics punctal plug delivery system has also been used with glaucoma drugs. It has been loaded with 95 *μ*g Latanoprost in Phase 2 clinical trials [209]. Intraocular pressure lowering at 4 weeks was measured as a primary endpoint with devices placed in the superior and/or inferior puncta for a 12 week period [210]. The ELUTYX technology has also been evaluated for release of travoprost in the canaliculus and with intracameral injection. A 90 d travoprost-releasing hydrogel-based implant in the canaliculus of the eyelid was evaluated in a Phase 3 trial. It demonstrated increased intraocular pressure lowering compared to placebo at 8 of 9 time points in a 3 month period but ultimately failed to meet the primary endpoint [209].

#### 5.3.3. Intracameral implantation

An ELUTYX-based intracameral hydrogel-based implant that delivers travoprost for a 6 month period completed Phase 1 clinical trials and is currently in development [211]. Two FDA-approved intracameral implants release prostaglandin analogs. DURYSTA^®^ (AbbVie) is an unanchored biodegradable bimatoprost-releasing intracameral implant. FDA approval for the 10 *μ*g dose of the implant was based on two Phase 3 clinical trials, which evaluated 3 implants at 16 week intervals over a 20 month period compared with topical timolol. While the 10 *μ*g dose was non-inferior, elevated rates of corneal and inflammatory events limited the approval to a single use. A later trial with a 15 *μ*g dose was associated with elevated rates of corneal adverse events, which halted clinical development [212].

The iDose^®^ (Glaukos) is an intracameral implant anchored in the trabecular meshwork. It contains 75 *μ*g of travoprost and is held in place by a biocompatible titanium cap. Drug release is managed with an ethylene-vinyl acetate membrane. In a Phase 3 clinical trial, a slow-eluting and fast-eluting implant were compared with topical timolol eyedrops. The slow-eluting implant was shown to be non-inferior to topical timolol for 12 months, while the fast-eluting implant was non-inferior for a period of 9 months [213].

#### 5.3.4. Intracapsular implantation

The lens capsule is accessible at the time of cataract surgery and offers a large volume for a drug delivery device. SpyGlass Pharma is developing a bimatoprost releasing insert (BIM-IOL) that is loaded on the haptics of a hydrophobic IOL for drug delivery during implantation. It is intended to deliver bimatoprost over 3 years and has been evaluated in Phase 1/2 clinical trials [214].

#### 5.3.5. Intravitreal implantation

PER-001 (Perfuse Therapeutics) is a biodegradable implant that has a selective endothelin receptor-A antagonist. It is made from a PLGA polymer matrix—-like the ones used with Ozurdex^®^ implants—-and is intended for a 6 month implantation. It acts via a non-intraocular pressure-dependent mechanism. PER-001 has been evaluated in Phase 1/2a clinical trials and is planned for a Phase 2b clinical trial [215].

While it is known that the modifiable risk factor of glaucoma is lowering intraocular pressure, most therapies have traditionally relied on eye drops [216]. Eye drops have significant side effects, including irritation and redness [217]. Further, non-compliance is common due to the burdensome nature of repeated eye drop regimens, side effects of treatment, and the often-lifelong necessity for therapy [217]. There are a number of extended drug delivery devices that aim to provide long-term delivery of glaucoma medications. The most common drugs used for this are prostaglandin analogs due to their potency and stability for long-term release formulation. For example, intraocular drug delivery systems with 75 *μ*g of prostaglandin analog have been shown to provide 3 years of drug delivery [218].

When drug delivery systems are not implanted into the eye, such as fornix- or contact lens-based drug delivery systems, the released drugs must overcome corneal barriers and loss from the tear film, which requires higher drug loading. For example, the bimatoprost ring required 2.5 mg over 6 months [205]. If this is delivered using zero order kinetics, this would correspond to 13.9 *μ*g d^−1^. For DURYSTA^®^ to release 10 *μ*g bimatoprost over 4 months with zero order kinetics, this would correspond to a daily release of 0.08 *μ*g d^−1^, which is 166 times less per day. Likewise, one drop of topical 0.1% bimatoprost daily is approximately 5 *μ*g d^−1^ of bimatoprost, or 60 times more than DURYSTA^®^. However, intraocular delivery requires a more invasive procedure for implantation; and therefore, involves higher surgical risks.

## 6. Emerging strategies and technologies in ocular drug delivery

Although successfully developed ocular drug delivery systems are currently in clinical use, recent research explores more complex and precise release mechanisms. This section will cover emerging ocular drug delivery implant research, including carrier improvements capable of stable payload delivery, exploration of new implantation locations, and development of stimulus-responsive systems.

### 6.1. Advances in stable therapeutic payload delivery

For stable payload delivery, several approaches have been introduced to improve the protection of implants *in vivo* or to maintain the original therapeutic’s structure and activity.

*Chemical surface modification* lowers surface energy and enhances lubrication to establish protective barriers for medical implants and improves biocompatibility to create a slippery interface. Such surfaces minimize droplet adhesion and cell attachment while preserving native device performance and biocompatibility. Wu *et al* presented a tannic acid (TA)-based zwitterionic polymer coating strategy on a glass surface to improve antibacterial and antifouling properties [219]. The zwitterionic polyethylenimine-quaternized derivative (PEIS)-functionalized antifouling coating process began with forming a silica sol layer on a glass substrate and then co-depositing TA and 3-aminopropyltriethoxysilane (APTES) to create a hydrophilic layer. The TA-coated substrate was grafted with a zwitterionic (PEIS) via Michael addition and Schiff-base reactions. The prepared substrate exhibited high hydrophilicity (contact angle 6.3°) but strong antibacterial activity and excellent antifouling performance (microalgae adhesion prevention >96%). Acosta *et al* developed a hybrid coating strategy using protein-engineered elastin-like recombinamers to conjugate antimicrobial peptides on the surface of implantable devices, owing to their ability to mimic the ECM [220]. This system showed superior antibiofilm activity against clinically relevant monospecies and microcosm biofilms and excellent cytocompatibility with human gingival fibroblasts, implying their potential to reduce the risk of implant-associated infections.

Separately, supercritical fluid technology, using supercritical CO_2_, was developed to ensure the drug’s original efficacy in the manufacturing process by eliminating the need for organic solvents or high temperatures. Bendicho-Lavilla *et al* developed dexamethasone and bevacizumab-loaded PLGA implants using supercritical foaming including a pressurization-soaking-single depressurization stepwise protocol [221]. They achieved different porosities depending on the depressurization rate, resulting in different drug release patterns. These implants also demonstrated sustained and controlled drug release for up to four months.

Genetic payloads have emerged as an advanced approach for treating inherited ocular diseases and provide the possibility for long-lasting or even curative outcomes. Despite their design flexibility, *in vivo* performances are limited by inefficient gene transfer, endosomal entrapment, and rapid degradation [222]. The next generation of gene delivery implants should surpass earlier designs in specificity, responsiveness, and clinical adaptability. In the eye, where multi-layered properties present unique physical and immune barriers, a single delivery approach is unlikely to be effective across the board. As a result, many studies have focused on developing biomaterials that can respond to local conditions and deliver therapeutic payloads to specific ocular cell types using precision-targeted systems. Ottonelli *et al* developed a thermo-sensitive hydrogel loaded with retina-targeted, hybrid nanoparticles for minimally invasive delivery to the retina [223]. Upon an intravitreal injection, the hydrogel underwent sol-gel transition, forming a biodegradable depot that enhanced drug loading and prolonged nanoparticle release with minimal toxicity and inflammation observed *in vivo*. Nishida *et al* further optimized this retinal-targeted hybrid nanocarrier system by incorporating a peptide embedded in a cationic liposomal matrix [224]. This hybrid system—–delivered topically as eye drops—-effectively penetrated ocular barriers and reached the retinal pigment epithelium (RPE), where it markedly improved cellular uptake and intracellular release of RNA. In a rabbit model, Kansara *et al* injected compacted DNA nanoparticles through a microneedle and achieved robust expression in both the retina and the RPE-choroid, and the procedure was well-tolerated [225]. This model supports microneedle-based suprachoroidal dosing as a minimally invasive way to pair the payload flexibility and lower immunogenicity of nonviral systems with broad posterior segment coverage. Story *et al* advanced the concept by introducing an intravitreal injection of copolymer microspheres embedded in a thermo-responsive hydrogel, providing months-long drug delivery in vitreous cavities of primates [226].

Encapsulated cell therapy biofactories use genetically engineered cells enclosed within semipermeable membranes to continuously secrete therapeutics. These systems provide a retrievable, long-term option. Clinical experience exists for CNTF platforms (e.g. NT-501) and anti-VEGF variants (e.g. NT-503), highlighting the importance of dose and secretion kinetics, manufacturing robustness, secretion stability, process consistency, and secure anchoring. To achieve a controlled biomass and regulation of CNTF secretion, Thanos and colleagues developed a microtexture-incorporated NT-501 for the treatment of retinitis pigmentosa [175]. The device consists of a poly(ether sulfone) membrane surrounding a scaffolding of poly(ethylene terephthalate) (PET) yarn loaded with a cell suspension and sealed with a urethane acrylate adhesive. Both PET and poly(butylene terephthalate) (PBT) yarns were subjected to alkaline hydrolysis, resulting in enhanced and prolonged viability [227].

### 6.2. Exploring new target sites

The suprachoroidal space is between the sclera and the choroid. It is situated outside the visual axis and adjacent to chorioretinal tissues. Although its small volume presents challenges in device implantation and retention, this location offers potential for high local exposure to the posterior segment while minimizing anterior-segment exposure. Targeted delivery of pharmaceuticals has been demonstrated using specialized suprachoroidal catheter systems [228]. Kim *et al* used gravity and positioning of the eye to drive high-density emulsion droplets to the macula in rabbit eyes. These high-density particle-stabilized emulsion droplets were constructed from a high-density perfluorodecalin surrounded by polystyrene nanoparticles, simulating pharmaceutical carriers [229]. Carboxymethyl cellulose can increase the viscosity of injections to keep them at the injection site for supraciliary injections [230]. Iontophoresis is applied to negatively-charged drug particles, driving them to the posterior portion of the eye after injection. A two-material approach is used to push injected pharmaceuticals to the posterior of the eye. A drug is first injected into the suprachoroidal space followed by injection of a swellable HA hydrogel, which expands [231]. This approach combines both components into a single injection with the hydrogel near the plunger and the drug at the needle end of the syringe. *In situ* hydrogels consisting of bevacizumab and HA crosslinked with PEGDA are used for extended drug delivery. Liquid formulations of the hydrogel are prepared and then injected into the suprachoroidal space. The initial low viscosity allows distribution throughout the suprachoroidal space, including the posterior segment, followed by *in-situ* gel formation for long-term sustained drug release of up to 6 months [232]. A light activated PCL dimethacrylate (PCM) and HEMA gel demonstrated extended suprachoroidal drug delivery of bevacizumab in a rat eye. Components of the gel are mixed, injected into the suprachoroidal space, and exposed to UV light for 10 min to cure [233].

### 6.3. Stimuli-responsive and actuated systems

Stimuli-responsive and actuated systems provide on-demand or externally controlled dosing and positioning. These implants can generally be divided into closed- and open-loop systems. Closed-loop systems rely on endogenous feedback, where an internal stimulus, such as a rise in intraocular pressure, increases drug release. Open-loop systems require an external stimulus, such as intended application of UV light or magnetic field, to achieve the on-demand desired response. To date, only open-loop systems have been explored for ocular drug delivery implants. Xia *et al* developed photo-responsive IOLs that can elute 5-Fluorouracil (5-FU) on demand, preventing posterior capsular opacification. They modified the surface of hydrophobic acrylic IOLs with reversible photodimerization and photolysis of coumarin and 5-FU. 5-FU can be released from the copolymer upon 254 nm of UV light. Upon irradiation, the coated IOLs prevent adhesion and proliferation of lens epithelial cells, which are related to posterior capsular opacification. The on-demand release relies on the UV light intensity and radiation time. *In vivo* assessments revealed that the developed IOLs reduced the occurrence of posterior capsular opacification after light irradiation with little side effects. Humayun and colleagues developed an intraocular micropump. This micropump delivery has entered Phase 1 clinical trials [147]. However, there is research into other mechanisms of action for an implantable micropump after trans-scleral implantation into the vitreous chamber of New Zealand White rabbits. The device consists of a 12.5 *μ*l reservoir, a MEMS-fabricated magnetic pump with a check valve system to prevent backflow [234]. To more efficiently target delivery through the vitreous, submicron micropropellers actuated by an external magnetic field were developed. They are fabricated using physical vapor shadow growth via glancing angle deposition with a silica structural element, nickel (or iron) magnetic segment, and perfluorosilane surface modification with perfluorocarbon layer and perfluorocarbon liquid layer. These devices were able to propel through the macromolecular porcine vitreous matrix by applying a rotating magnetic field [235].

### 6.4. Technical bottlenecks and remaining challenges to be overcome

Despite the potential for genetic payloads, the effective application of gene-therapy implants remains limited by persistent delivery challenges. One of the challenges is ensuring the safe, efficient, and durable delivery of therapeutic genetic material to ocular cells. Immune-mediated clearance of vectors or carrier systems [236], suboptimal transduction efficiency in critical ocular cell types (e.g. photoreceptors, RPE) [237], and temporary therapeutic benefit from implant-based platforms [238] continue to compromise therapeutic efficacy. Such obstacles will require further integration of biomaterial designs, unique targeting strategies, and optimized delivery routes to fully realize the therapeutic potential of gene-therapy implants in ocular disease.

Encapsulated cell therapy biofactories show promise for long-term drug delivery. However, there are challenges with this technology. Controlling the amount of drug made may be variable between devices, or between benchtop and *in vivo* conditions. Further, producing enough of a drug for effective therapy can be challenging, as seen with NT-503 [180, 181, 239]. Cells themselves produce more than just the pharmaceutical factor, and other secreted factors may interact in positive or negative ways [240]. The types of drugs produced are limited to what cells can produce and fold effectively at high concentration. This often limits cells to producing certain protein molecules. The structure of the device includes a semipermeable membrane to protect the encapsulated cells from immune attacks. However, this semi-permeable membrane may limit or prevent the release of certain drugs [241]. Overcoming these challenges to produce enough of a drug for effective therapy remains difficult. This has been overcome for CNTF in macular telangiectasia but must be addressed for other drugs in other diseases [138].

Targeted drug delivery in the suprachoroidal space provides a new technique for managing retinal disease. While light-activated and *in situ* gel techniques have been developed in animal models, the reliability and safety of these techniques have not been established in humans [229–233]. For UV based curing, the safety of UV-activated light gels in humans and possible ocular or retinal toxicity needs to be further addressed [242]. Other challenges for hydrogels include shelf-life, sterilization, scale-up, and user compliance by clinicians and/or patients [4].

Stimuli-responsive and actuated systems offer hope for truly custom therapies of multiple drugs for patients. However, drugs need to be stable in the reservoir for prolonged periods of time without degradation [243]. Further, long-term implantation of reliable complex mechanical and electromechanical devices can be challenging in terms of manufacturing and design [244, 245]. The use of magnetically actuated micro- and nano-devices has expanded to address the challenges of navigating the dense, mesh-like, non-Newtonian structure of the vitreous, where penetration through such viscous tissues can be limited [246]. Achieving effective propulsion and steering in this complex biological network remains an active research area, as the heterogeneous viscosity and structural obstacles of dense extracellular matrices can influence micropropellers motion and navigation. In addition, most studies so far are conducted *in vitro*, and only a few systems have been able to combine real-time *in vivo* imaging [247], because tracking, actuation, and monitoring drug release inside living tissue are still difficult. Submicron micropropellers may increase the efficacy of drug delivery. A perfluorocarbon molecule is coated with a low-friction molecule to overcome challenges of adhesion between collagen fibrils in the vitreous and the micropropellers. Further challenges include improving directionality, potentially using controlled injections and real-time feedback navigation [235]. In addition, long-term biocompatibility with Ni or Fe needs to be addressed, along with biodegradability. Long-term tissue interaction and retinal toxicity *in vivo* also need further research. For deployment to patients, delivery systems and peripheral devices to guide therapy need to be developed, as well as treatment schedules and protocols. For example, delivery from the center of the vitreous to the retina currently takes 30 min [235]. How this will be performed practically, or whether time can be reduced, will need to be further investigated. Finally, MRI compatibility with such devices is an open issue [244].

## 7. Future directions

Along with the current direction of prioritizing complex and targeted treatment, future research will pursue *personalized ocular drug delivery implants*. Patient-specific systems with optimized dosing profiles and depot distributions can be designed based on patient-derived genetic and geometric information. Therapeutic responses, complication occurrences, and clearance profiles can be estimated by machine learning technology. Personalized ocular implants will lead to highly efficient treatments.

In parallel, *improved material manufacturing* is vital to enhance the efficacy of implants. Advances in anti-fouling systems made of zwitterionic or poly-para-xylylene, PLGA grafts engineered to yield neutral byproducts, and real-time tunable hydrogels responsive to ocular tissue properties all make implants more reliable. Beyond conventional gene delivery strategies, emerging on-demand manufacturing platforms such as RNA printers introduce a new wave of RNA innovation for controlled gene therapeutics. With the development of automated cell-free RNA printers [248] and microneedle vaccine printers using lipid nanoparticles loaded with mRNA in dissolvable polymers [249], RNA could potentially be produced and delivered on demand in a precise manner for vaccines and therapeutics without relying on plasmid DNA templates. However, major challenges are due to its general susceptibility to degradation during and after printing, the need for compatible, biologically inert bioinks that preserve RNA integrity, and the development of delivery modalities that ensure effective cellular uptake without triggering immunogenic responses. In addition, it is important to address scalability and manufacturing reproducibility to transition to clinical applications, considering factors like gene therapeutic impurities, standardized rapid release, and potency assays under a specific fabrication environment.

Indent of ocular implants will rely on *addressing regulatory and commercialization challenges*, which are easy to overlook in the development stage. Specific requirements can vary depending on the device, so engagement with regulatory agencies is beneficial to increase approval probabilities and reduce total development-to-translation costs. Newly developed implants, materials, or manufacturing approaches may require new or updated guidelines and standards. Furthermore, scalable manufacturing (in terms of productive yields) and simplifying clinical use for surgeons in the operating room should also be considered early on. Taken together, advances in personalization, materials, regulation, and scalability will lead to durable, safe, and reliable ocular therapies.

## Figures and Tables

**Figure 1.**
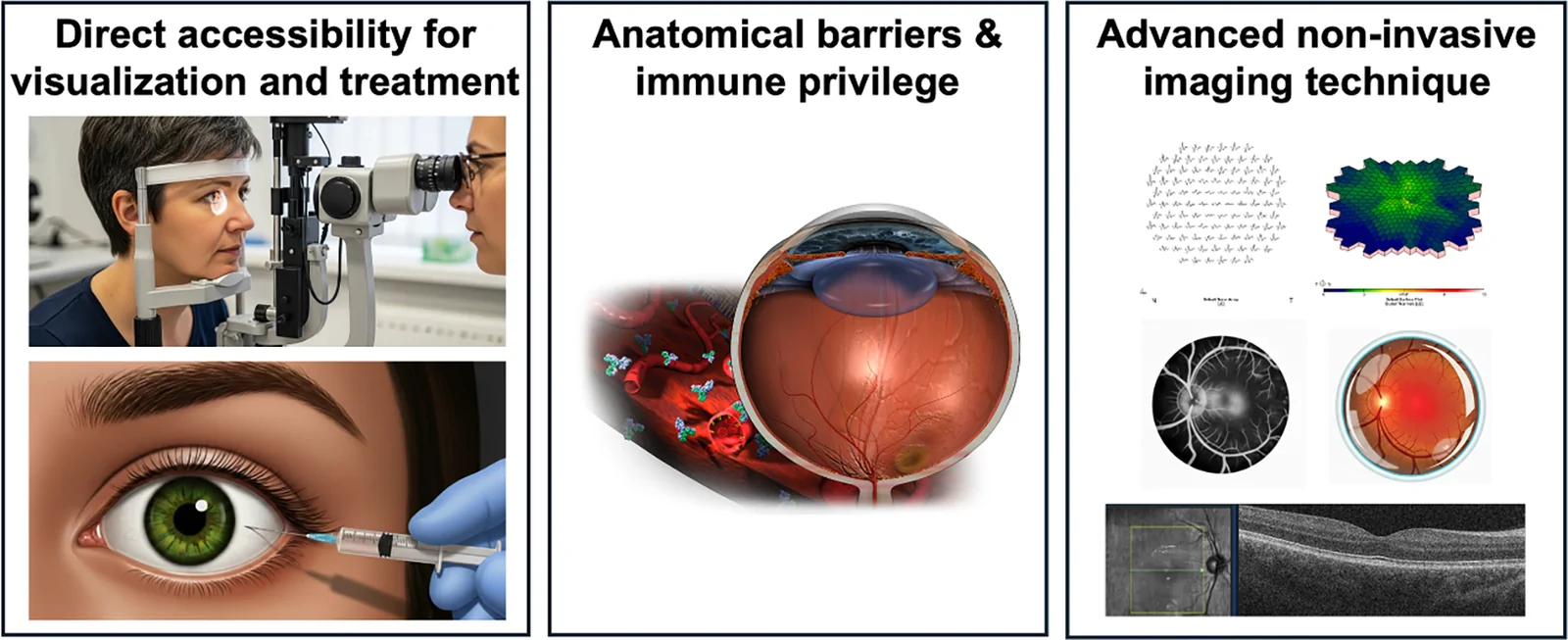
Advantages of the eye for biofabricated implants. The images in the boxed panels titled ‘Direct accessibility for visualization and treatment’ and ‘Advanced non-invasive imaging technique’ were created with Whisk Labs (experimental version; model: Imagen 4; Mid-to-Late 2025 release; source: whisk.ai).

**Figure 2.**
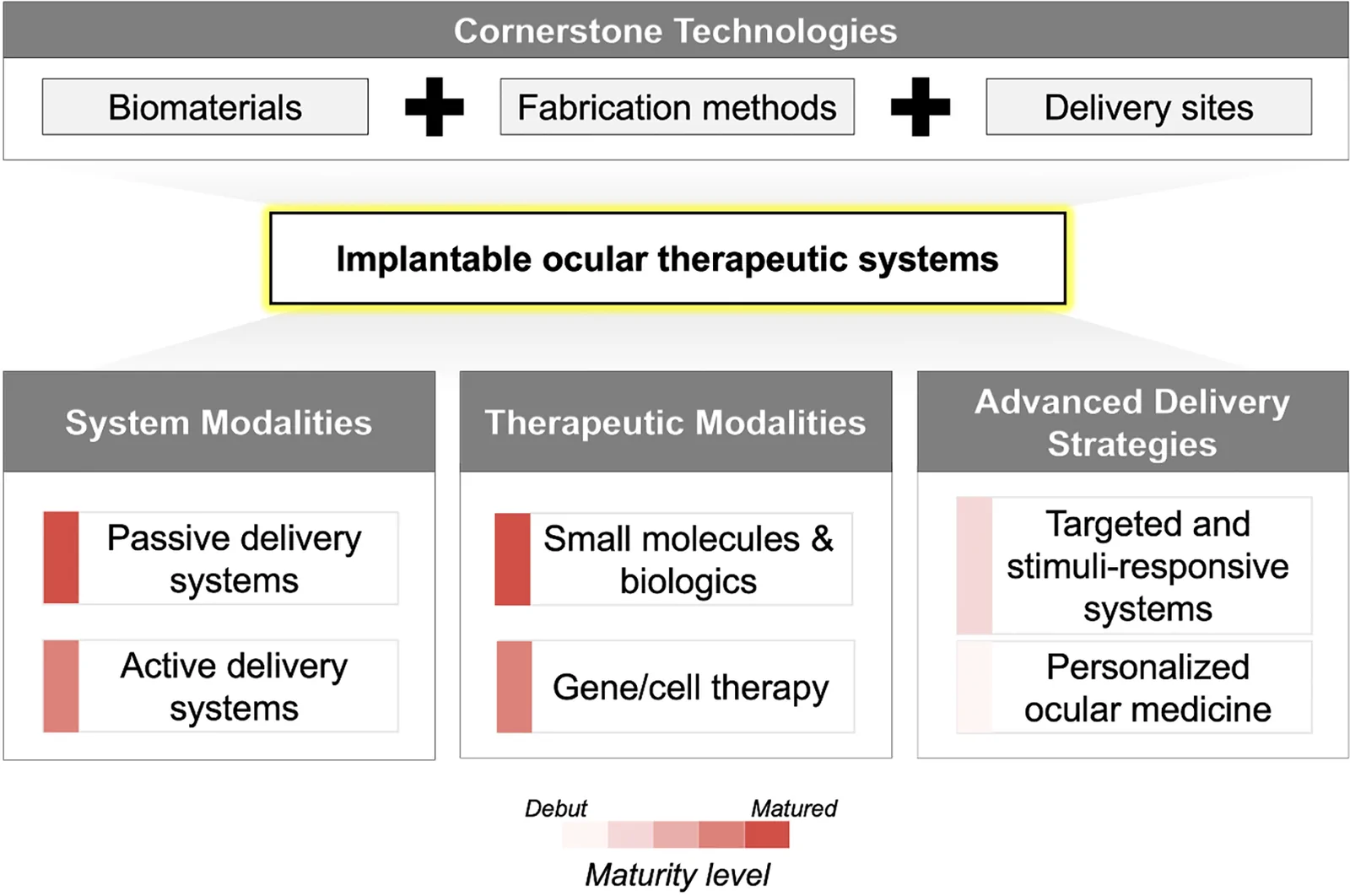
Schematic overview of the development of implantable ocular therapeutic systems.

**Figure 3.**
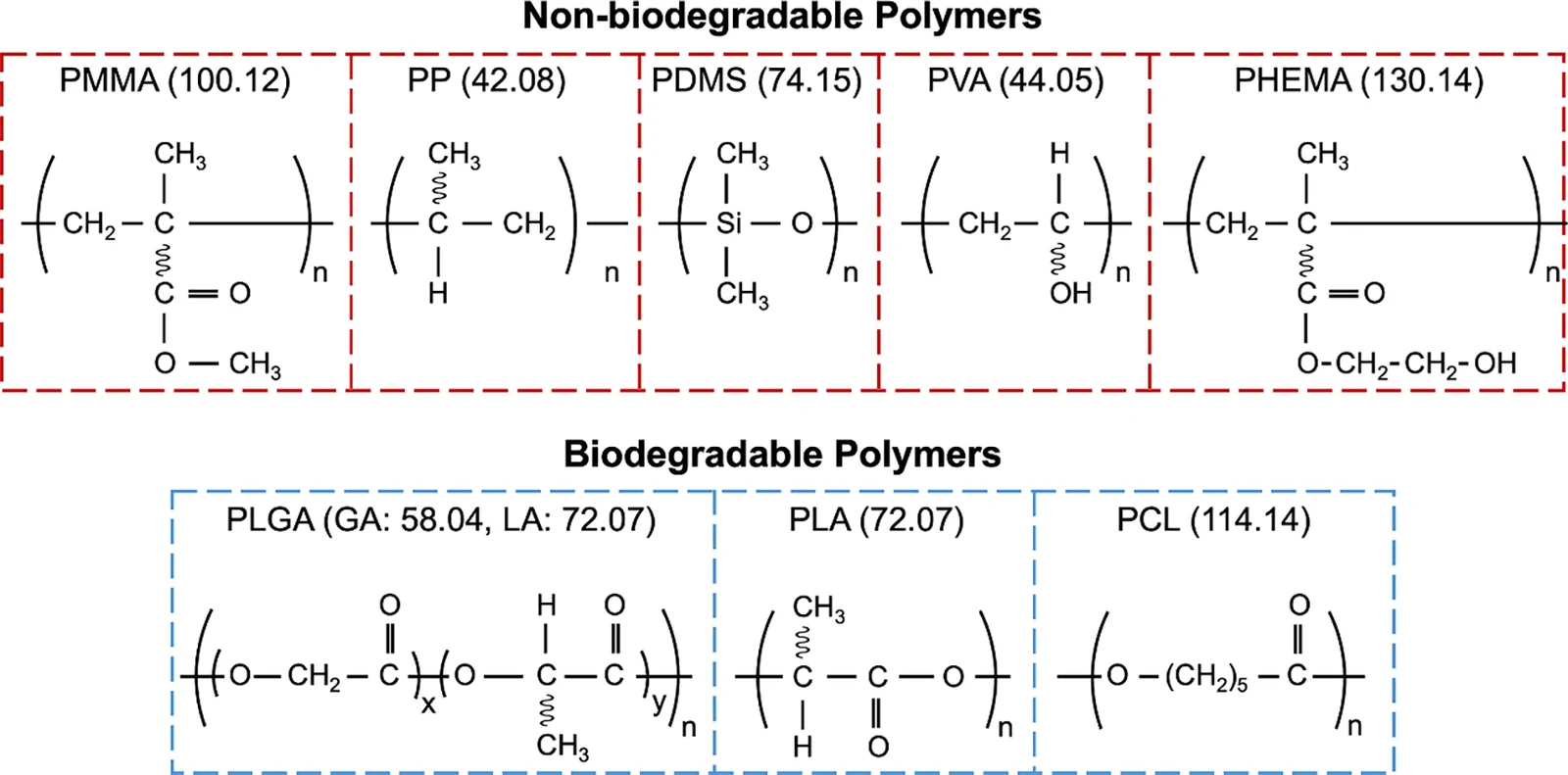
Chemical structures of representative monomers forming the most widely used synthetic polymers in ocular drug delivery. Wavy bonds indicate unspecified or atactic stereochemistry. The molar mass of each monomer is indicated in g mol^−1^. Abbreviations: PMMA, polymethyl methacrylate; PP, polypropylene; PDMS, polydimethylsiloxane; PVA, polyvinyl alcohol; PHEMA, poly(2-hydroxyethyl methacrylate); PLGA, poly(lactic-co-glycolic acid); GA, glycolic acid; LA, lactic acid, PLA, polylactic acid; PCL, poly(caprolactone).

**Figure 4.**
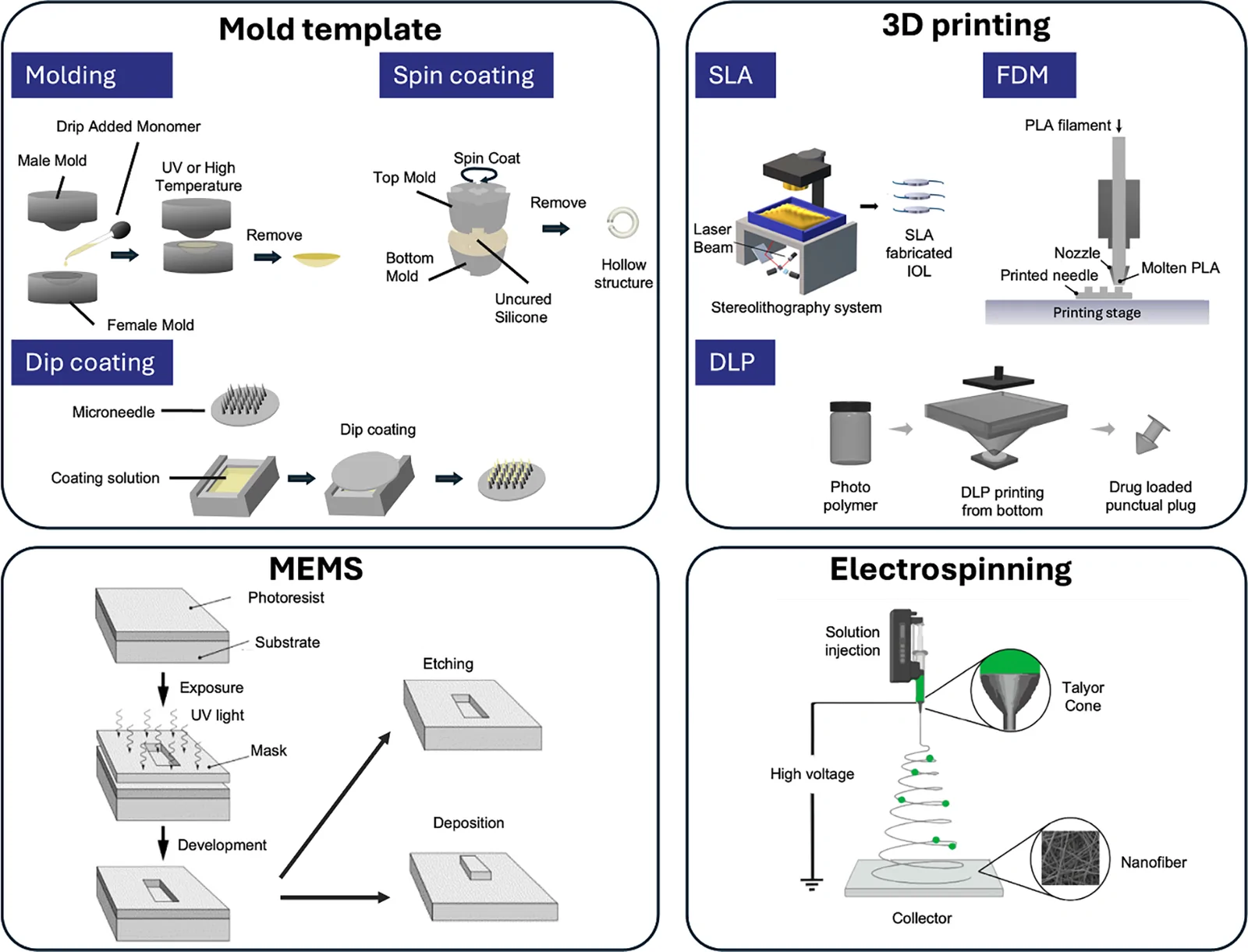
Various fabrication methods for ophthalmic therapeutic implants. (a) Molding (Reprinted from [63], Copyright (2016), with permission from Elsevier), spin coating (Reprinted with permission from [64]. Copyright (2025) American Chemical Society), and dip coating (Reprinted from [65], Copyright (2019), with permission from Elsevier) for different structures, such as intraocular lens, hollow drug reservoirs and drug coated microneedles. (b) stereolithography (SLA, Reproduced from [66]. CC BY 4.0), fused deposition modeling (FDM, Reproduced from [67]. CC BY 4.0), and digital light processing (DLP, Reproduced from [68]. CC BY 4.0) 3D printing methods under different conditions like temperature laser for different materials. (c) Micro-electro-mechanical systems (MEMS, Reprinted from [69], Copyright (2003), with permission from Elsevier) fabrication includes lithography, deposition, and etching to create fine features. (d) Electrospinning creates permeable structures using high voltage (Reproduced from [70]. CC BY 4.0).

**Figure 5.**
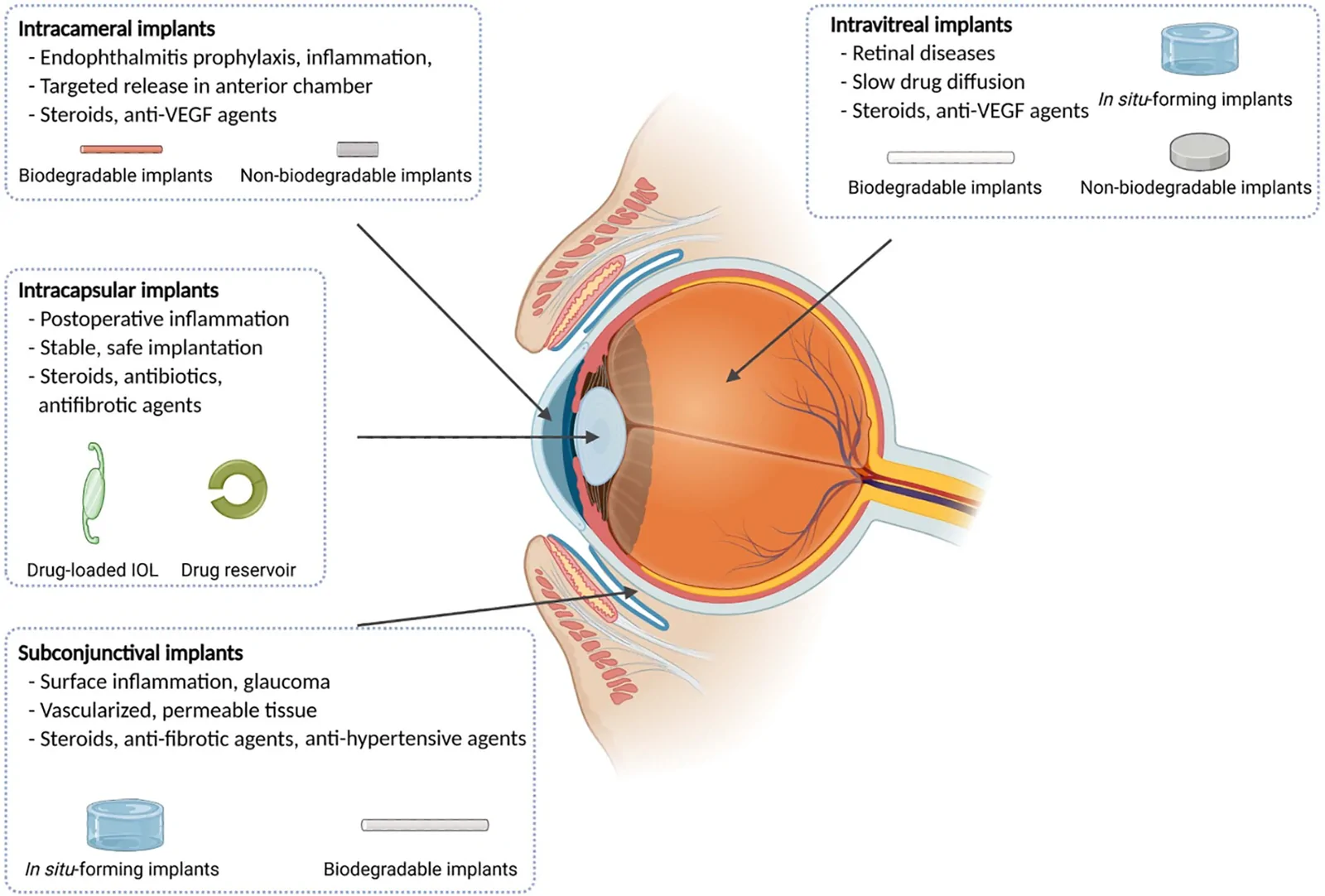
Site-specific strategies for ocular drug delivery implant design. Created in BioRender. Jang (2026) https://BioRender.com/pdmussj.

**Figure 6.**
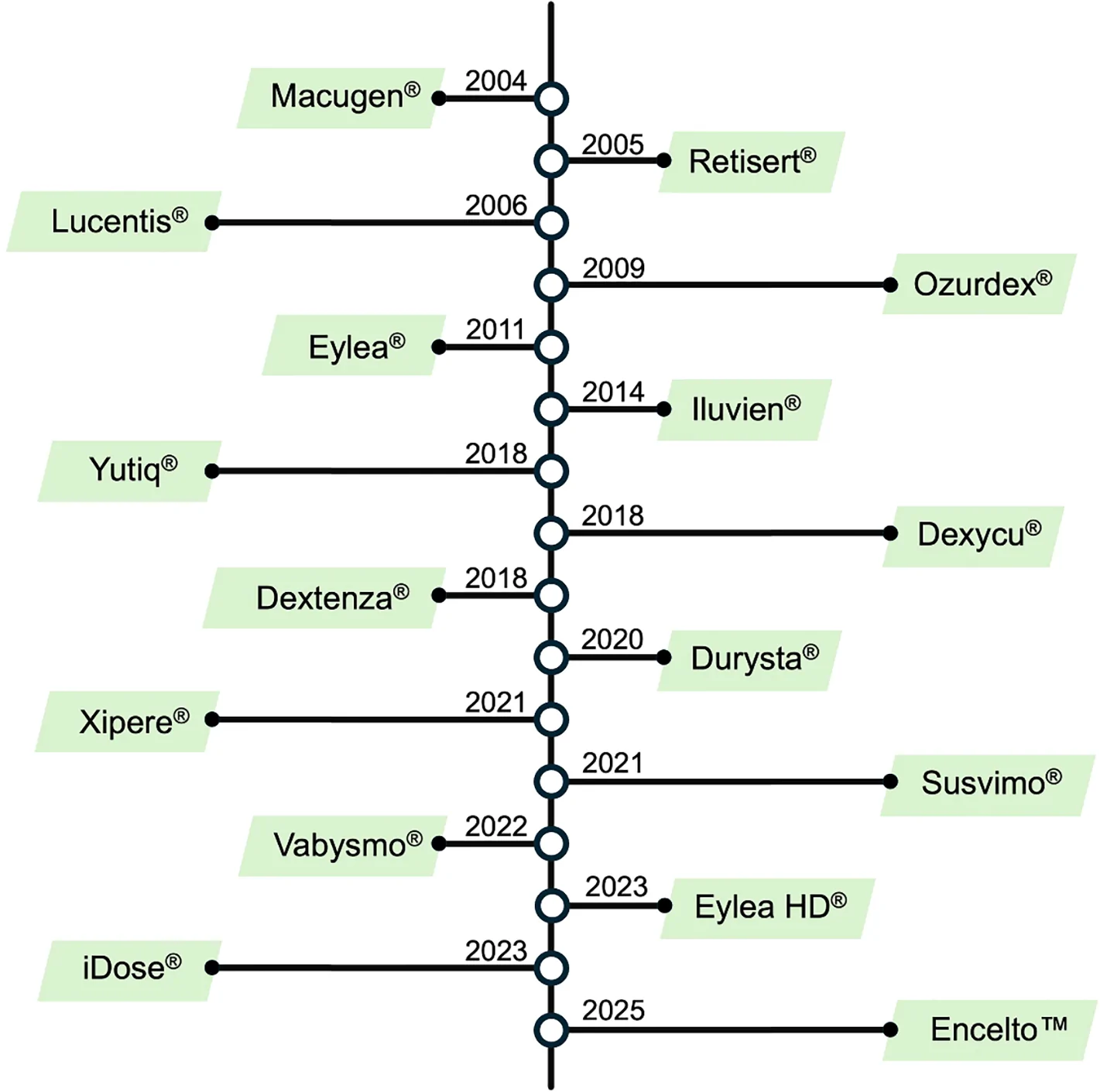
Timeline of ophthalmic therapeutic implants and their FDA approval dates.

**Figure 7.**
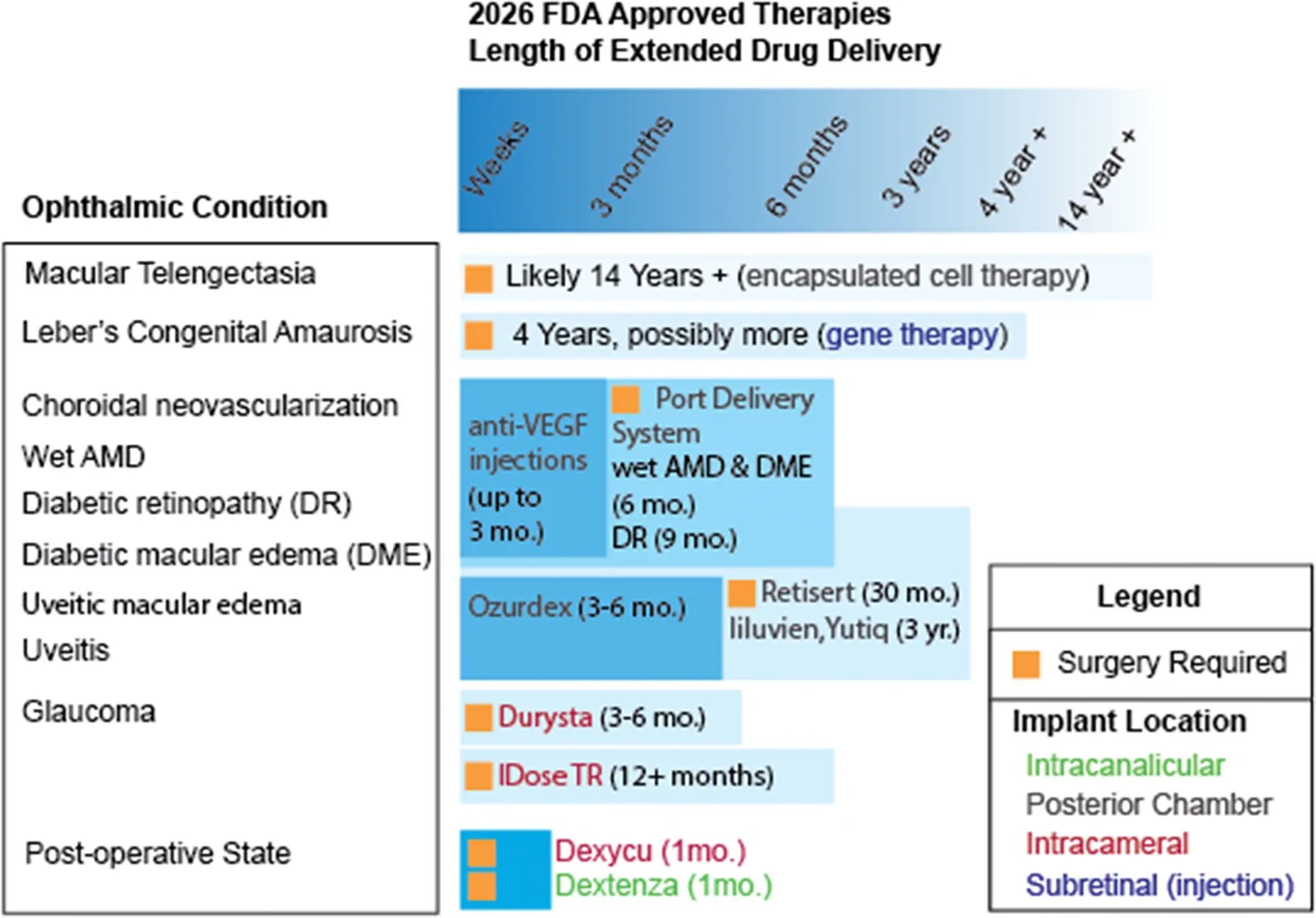
FDA-approved ophthalmic therapies as of 2026, categorized by ophthalmic condition and duration of extended drug delivery.

**Table 1.** Key synthetic polymers for ocular drug delivery and their evaluative comparison. Abbreviations: PMMA, polymethyl methacrylate; PP, polypropylene; PVA, polyvinyl alcohol; PHEMA, poly(2-hydroxyethyl methacrylate); PLGA, poly(lactic-co-glycolic acid); PLA, polylactic acid; PCL, poly(caprolactone).

Polymer (class)	Degradation profile	Key mechanical/optical features	Drug-loading & processing considerations	Major limitations

PMMA (thermoplastic)	Nondegrading; long-term residence unless explanted	Very stiff (Young’s modulus ≈2 GPa), minimal water uptake, optically clear with refractive index ≈1.49	High temperature molding (*T_g_* ≈ 105 °C–113 °C) prevents direct loading of most heatable drugs; mainly used as inert structural carrier or coating	Requires surgical removal if problematic; hydrophobic surface can adhere to tissues; not well suited as a primary drug release matrix
PP (thermoplastic)	Non-degrading; permanent unless removed	High strength, semicrystalline, Young’s modulus ≈1–2 GPa, opaque or translucent rather than optically clear	Melt extrusion and molding at ≈160 °C; not compatible with direct drug encapsulation during processing; useful mainly as mechanical support	No optical clarity; hydrophobic, poor as a drug diffusion matrix; surgical burden for implantation and explantation
Silicone (elastomer)	Nondegrading; stable long-term *in vivo*	Soft, elastomeric (modulus hundreds of kPa to a few MPa), highly flexible, optically clear with refractive index ≈1.42–1.43	Two-part systems, cured at ⩽100 °C; enables embedding of lipophilic drugs; release often needs rate limiting membranes to avoid burst	Requires surgical implantation and removal; hydrophobic matrix favors lipophilic drugs; control of initial burst and long-term kinetics can be challenging
PVA (hydrogel)	Crosslinked networks are non-degrading; persistent unless explanted	High water content (≈70%–80%), transparent (refractive index ≈1.33–1.35), modulus ≈0.1–2 MPa depending on crosslinking	Aqueous, low temperature processing allows premixing or post loading of drugs; supports relatively fast diffusion and often shows burst release	Requires removal if long-term presence is undesired; relatively weak mechanics; rapid diffusion and burst release unless network is modified
PHEMA (hydrogel)	Non-degrading hydrogel; chronic presence unless removed	Soft hydrogels with 38%–70% water; modulus ≈0.1–0.8 MPa; optically clear and foldable	Cast molded and hydrated; open network allows hours to days release; strategies like nanoparticle doping or imprinting needed to extend release	Limited oxygen permeability vs silicone hydrogels; relatively short drug release durations without additional engineering
PLGA (polyester)	Weeks-months; tunable with lactide:glycolide ratio and Mw (e.g. ∼3–6 months for Ozurdex^®^)	Amorphous, relatively stiff and brittle initially (modulus ≈1–2 GPa), softens as it hydrates and erodes	Solvent-based casting, extrusion, and emulsion-based microspheres with embedded drug; suitable for many small molecules and biologics	Acidic degradation products can lower local pH and induce inflammation; anterior segment use (e.g. intracameral bimatoprost) shows endothelial risk with repeat dosing, limiting redosing frequency
PLA (polyester)	Slow, multi-month to multi-year bulk erosion (≈6–24 months depending on formulation)	Hydrophobic; higher mechanical strength than PLGA; semi-crystalline (PLLA) or amorphous (PDLLA)	Often processed via solvent casting or low temperature extrusion; suitable for long-lasting scaffolds and slower drug release	Persistent fragments can provoke size-dependent foreign body responses; degradation is slow, so long-lived remnants must be considered in ocular compartments
PCL (polyester)	Very slow degradation; months to ⩾1 year with prolonged implant persistence	Semicrystalline, flexible at body temperature; modulus in the hundreds of MPa, elongation 300%–500%	Low melting point (≈60 °C) supports melt processing and 3D printing with drug; strongly favors hydrophobic drug loading and very prolonged release	No approved PCL ocular drug product yet; hydrophilic drugs can crystallize and leach rapidly; extremely slow degradation demands careful evaluation of chronic tolerability

**Table 2.** Key fabrication methods used for ocular drug delivery and their evaluative comparison. Abbreviations: MEMS, micro-electro-mechanical systems.

Fabrication method	Resolution	Scale- fabrication	Advantages	Limitations	Other properties

Mold templates	Relatively low (∼hundreds of microns)	Yes	Broad material compatibility; high throughput and low unit cost; good part-to-part consistency	Not suitable for very fine or highly complex internal features; trapped bubbles and voids, polymer shrinkage	Material selection and pretreatment may be required for demolding
3D printing	Relatively low (∼tens of microns)	Limited, but yes	High design freedom to complex structures; good customization	Struggling with sub-100 μm resolution and surface roughness; material limitation	Suitable for customized requirements and complex geometry
MEMS	Relatively high (∼1–5 μm)	Limited, but yes	High precision; reliability and batch repeatability	Process biocompatibility	Suitable for miniaturization requirements
Electrospinning	Relatively high (10 nm—several microns)	No	High surface area; highly porous structure	Low productivity, production inconsistency, difficulty forming complex shapes, and safety issues	Provides high surface area, porosity

**Table 3.** Ocular therapeutic devices and their evaluative comparison. Abbreviations: DME, diabetic macular edema; AMD, age-related macular degeneration; MEMS, micro-electro-mechanical systems.

Device	Company	Target disease	Fabrication method	Pros	Cons	Status
Posterior MicroPump drug delivery system	Replenish, Inc.	DME	MEMS	1.Patient specific drug delivery 2. Telemetry 3. Potential to deliver many categories of drugs	Complex electromechanical design	First-in-human safety study complete
Port delivery system	Genentech	Wet AMD; Diabetic Retinopathy; DME	Over molded septum with epoxy bonding	1. Established long-term delivery of Anti-VEGF molecules 2. Expandable to other pharmaceuticals 3. Iterated and improved design	1. Surgical implantation complexity 2. Initial elevated rates of infection, vitreous hemorrhage, and erosion of conjunctiva	FDA approved
Vitrasert^®^	Chiron Corporation/Bausch and Lomb/pSivida Ltd.	Cytomegalo-virus retinitis	Coating process of drug pellet, connected to a suture strut	1. Long-term anti-viral drug delivery	Requires surgical implantation	Discontinued
Retisert^®^	Bausch and Lomb	Chronic non-infectious uveitis	Coating process of drug pellet, with silicone elastomer cup bonded to a suture strut using a silicone adhesive	1. Long term steroid delivery at relatively high levels	1. Requires surgical implantation 2. Side effects of steroid drugs	Planned discontinuation in 2026
Iluvien^®^ and Yutiq^®^	ANI Pharmaceuticals	DME; Non-infectious uveitis		1. Injectable without requiring surgery 2. 3 year drug delivery	1. Non-biodegradable 2. Side effects of steroid drugs	FDA approved
Ozurdex^®^	Allergan Pharmaceuticals	DME or macular edema from vein occlusion; Non-infectious uveitis	Hot-melt extrusion	1. Injectable without requiring surgery 2. Biodegradable	Shorter delivery time than Iluvien^®^	FDA approved
I-vation^™^	Surmodics	DME	Coiling a titanium wire; Laser-welding the cap; Proprietary coating process	Long-term steroid delivery	1. Side effects of steroid drugs	Phase 2 terminated
Encelto^™^	Neurotech	Macular Telangiectasia Type 2	Encapsulated transgenic cells inside a semipermeable membrane	Long-term drug delivery for years	1. Surgical implantation required 2. Potential limitations regarding which drugs can be delivered and delivery rates	FDA approved
BRIMO DDS^®^	Allergan/AbbVie	Geographic Atrophy	Brimonidine free base loaded in polymer matrix	Intravitreal injection	Limited efficacy for geographic atrophy	Terminated Phase 2b trial
Duravyu^™^	EyePoint Pharmaceuticals	Wet AMD; Diabetic Retinopathy; DME	Vorolanib embedded in matrix	1. Intravitreal injection 2. Bioerodible 3. 6 mo. delivery		In FDA clinical trials
Axplaxi^™^	Ocular Therapeutix	Wet AMD; Diabetic Retinopathy	Micronized axitinib formulated with polyethylene glycol	1. Intravitreal injection 2. Bioerodable 3. 9–12 mo. delivery		In FDA clinical trials
AXT107	AsclepiX Therapeutics	Wet AMD; DME	Self-assembles into an *in-situ* gel after injection	Intravitreal or suprachoroidal injection	Potential for visual disturbance if intravitreal injection in visual axis	In FDA clinical trials
DEXYCU^®^	EyePoint Pharmaceuticals	Postoperative State		Injected at time of surgery		FDA approved
Dextenza^®^	Ocular Therapeutix	Postoperative State		Inserted at time of surgery		FDA approved
Evolute^®^ PPDS	MATI Therapeutics	Postoperative State; Glaucoma		Does not require surgical insertion		In FDA clinical trials
Bimatoprost Ring	Allergan/AbbVie	Glaucoma		Does not require surgical insertion	1. Efficacy compared to standard treatment 2. Comfort and tolerability of conjunctival insert	Had a Phase 2 FDA clinical trial
Topical ophthalmic drug delivery device	Amorphex Therapeutics	Glaucoma; Myopia	Cast molding	Does not require surgical insertion	Comfort and tolerability of conjunctival insert	Tolerability in humans
Eye-D	BioLight Life Science	Glaucoma			Subconjunctival insertion maybe more involved than a conjunctival insert	Evaluated in Phase 1/2a trial
Durysta^®^	Abbvie	Glaucoma		Simple insertion	Corneal endothelial damage limits re-use	FDA approved
iDose^®^	Glaukos	Glaucoma		1. Relatively long-term drug delivery	1. Surgical insertion requires significant skill and training	FDA approved
BIM-IOL	SpyGlass Pharma	Glaucoma		Implanted at time of cataract surgery	Requires cataract surgery to implant	Evaluated in Phase 1/2 clinical trial
PER-001	Perfuse Therapeutics	Glaucoma		1. Intravitreal insertion 2. Bioerodible		Evaluated in Phase 1/2 clinical trial

## Data Availability

No new data were created or analyzed in this study.
